# Mass Spectrometry-Based Proteomics Methods for Systematic Identification and Quantification of Protein O‑Glycosylation in Complex Biological Samples

**DOI:** 10.1021/jasms.6c00005

**Published:** 2026-04-03

**Authors:** Longping Fu, Xing Xu, Ronghu Wu

**Affiliations:** School of Chemistry and Biochemistry and the Petit Institute for Bioengineering and Bioscience, 1372 Georgia Institute of Technology, Atlanta, Georgia 30332, United States

**Keywords:** Bottom-up Proteomics, Enrichment Methods, Fragmentation, Intact O-Glycopeptides, O-Glycoproteomics, Mass Spectrometry

## Abstract

Protein O-glycosylation is one of the most common and important modifications in human cells. It regulates protein folding, trafficking, stability, and interactions with other molecules, and its dysregulation is directly related to numerous diseases such as cancer and neurodegenerative diseases. Modern mass spectrometry (MS)-based proteomics provides a unique opportunity to systematically characterize O-glycosylated proteins. However, it is still extremely challenging due to the low abundance of many glycoproteins, the heterogeneity of O-glycans, and the complexity of biological samples. In this review, we discuss recent advances in MS-based proteomics methods designed to overcome the challenges for global and site-specific characterization of protein O-glycosylation. We begin with an overview of the biosynthetic pathways underlying the major classes of protein O-glycosylation. Then, we discuss different methods to enrich O-glycopeptides with diverse structures of O-glycans. Furthermore, various MS dissociation techniques for intact glycopeptide profiling are covered. In addition, different quantitative approaches are included for studying protein O-glycosylation in biological and biomedical research. We also discuss computational tools for intact O-glycopeptide identification, highlighting the challenges in search space requirement, false discovery rate control, and glycosylation site localization. The advancements of MS-based glycoproteomics are critical for gaining insights into the critical roles of protein O-glycosylation in biology and human disease.

## Graphical abstract

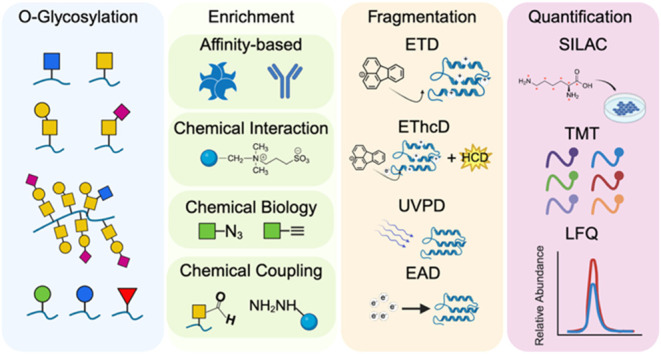

## Introduction

Protein glycosylation is extremely important in cells because it regulates many cellular events, such as cell immune response and cell–cell communication.^1–3^ Protein glycosylation is a nontemplated process, orchestrated by hundreds of enzymes, which results in a remarkable diversity of glycan structures and multiple types of amino acid residues being glycosylated. Dysregulation of protein glycosylation is directly related to human diseases, including cancer and inflammatory disorders.^4,5^ There are two major types of protein glycosylation, i.e., N-glycosylation, the attachment of glycans to asparagine (Asn) residues, and O-glycosylation, which primarily occurs on serine (Ser) and threonine (Thr) residues. N-Glycosylation features a conserved core structure, whereas O-glycosylation can be initiated by various monosaccharides, including N-acetylgalactosamine (GalNAc), N-acetylglucosamine (GlcNAc), mannose (Man), glucose (Glc), and fucose (Fuc).^6^ Protein N-glycosylation and mucin-type O-glycosylation are most common, and they are predominantly associated with the cellular classic secretory pathway, while O-GlcNAcylation mainly occurs in the nucleus and the cytoplasm, and is critical for the regulation of gene expression and cellular signaling.^7^

Mass spectrometry (MS)-based proteomics has emerged as a very powerful method for investigating proteins and their modifications including glycosylation.^8–18^ However, global analysis of protein glycosylation in biological samples is still very challenging due to the low abundance of many glycoproteins, the heterogeneity of glycans, and the complexity of biological samples.^3,19,20^ Unlike N-glycosylation, protein O-glycosylation (including both O-GlcNAcylation and mucin-type O-glycosylation) normally lacks a defined sequence consensus motif, making the precise localization of glycosylation sites even more difficult. Furthermore, the dense glycan cluster on mucin-type glycoproteins renders them highly resistant to proteolytic digestion, limiting their characterization using conventional bottom-up proteomic workflows.^21^ Therefore, developing new methods to overcome these hurdles for analyzing protein O-glycosylation is urgently needed.

In this review, we focus on MS-based methods that address the challenges of O-glycoproteomics analysis in complex biological samples. We first provide an overview of the biosynthetic pathways and structural diversity of major types of protein O-glycosylation. We then highlight recent advances in enrichment methods for capturing glycopeptides with different types of O-glycosylation, followed by multiple MS fragmentation approaches for intact glycopeptide characterization. In the last part, we discuss the establishment of quantitative workflows and computational tools to investigate protein O-glycosylation in complex biological samples. Given the scope and ongoing expansion of the field, only relatively more relevant papers are included in this review, and it is impossible to have an exhaustive survey.

### Biosynthetic Pathways and Diversity of Protein O-Glycosylation

Protein O-glycosylation encompasses different types of glycosylation in which glycans are attached to the hydroxyl group of serine (Ser), threonine (Thr) and tyrosine (Tyr) residues. Unlike N-glycosylation, which requires the Asn-X-Ser/Thr consensus sequon and proceeds through a conserved lipid-linked oligosaccharide precursor, O-glycosylation is initiated by the direct transfer of monosaccharides to the polypeptide backbone without a universal precursor or consensus motif.^6^ Multiple structurally distinct classes have been described in mammals, each initiated by different glycosyltransferases and localized to specific subcellular compartments (Figure 1).^6,22^

**Figure 1:**
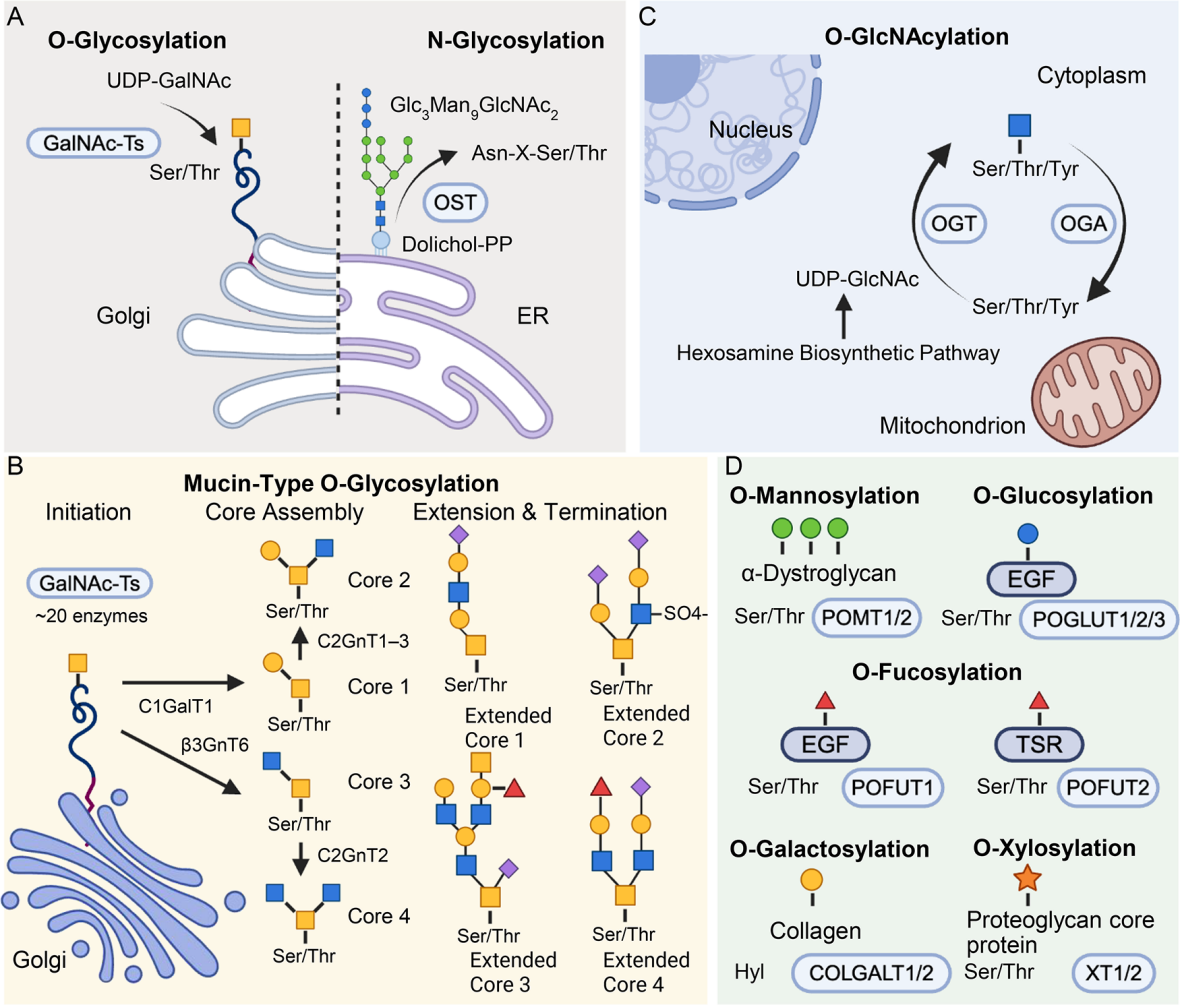
Biosynthetic pathways and structural diversity of the major classes of protein O-glycosylation. (A) Subcellular localization of mucin-type O-glycosylation initiation in the Golgi, shown alongside N-glycosylation in the ER for comparison. (B) Mucin-type O-glycosylation core structure assembly (cores 1–4), extension by poly-N-acetyllactosamine chains, and termination by sialic acid or fucose. (C) Dynamic O-GlcNAcylation cycling between OGT and OGA on nuclear, cytoplasmic, and mitochondrial proteins, with UDP-GlcNAc supplied through the hexosamine biosynthetic pathway. (D) Domain-specific O-linked modifications, including O-mannosylation (POMT1/2, α-dystroglycan), O-fucosylation (POFUT1, EGF-like repeats; POFUT2, thrombospondin type 1 repeats), O-glucosylation (POGLUT1/2/3, EGF-like repeats), O-galactosylation (COLGALT1/2, collagen hydroxylysine), and O-xylosylation (XT1/2, proteoglycan core proteins).

Mucin-type O-glycosylation is the most prevalent and heterogeneous form. It is initiated in the Golgi by up to 20 polypeptide GalNAc-transferases (GalNAc-Ts), which transfer GalNAc from UDP-GalNAc to Ser/Thr.^23^ Individual GalNAc-T isoenzymes exhibit partially overlapping but nonredundant substrate specificities, with the subfamily I members recognizing unmodified peptide substrates and the subfamily II members preferentially glycosylating GalNAc-bearing glycopeptides, enabling hierarchical site occupancy.^23^ The glycan is further elaborated to form four common core structures: core 1 (Galβ1–3GalNAc, T antigen) formed by C1GalT1 (requiring the ER chaperone Cosmc); core 2, produced by branching via C2GnT1–3; core 3 (GlcNAcβ1–3GalNAc) generated by β3GnT6; and core 4, from further branching of core 3 by C2GnT2.^24^ These cores can be extended by poly-N-acetyllactosamine chains and terminated by sialic acid or fucose. Premature sialylation of the Tn antigen by ST6GalNAc-I produces the cancer-associated sialyl-Tn (STn) epitope, a truncated structure reflecting dysregulated biosynthetic processing.^25^ Four additional rare cores (5–8) have been identified, though their biosynthetic enzymes remain incompletely characterized.

O-GlcNAcylation is fundamentally distinct in subcellular localization and biological functions. It is a dynamic, reversible modification of nuclear, cytoplasmic, and mitochondrial proteins, cycling between the addition of the glycan by O-GlcNAc transferase (OGT) and the removal of the glycan by O-GlcNAcase (OGA).^24^ OGT uses UDP-GlcNAc produced through the hexosamine biosynthetic pathway, making O-GlcNAcylation a metabolic sensor integrating nutrient status with signaling.

O-Mannosylation, mediated by POMT1/2 in the ER, is best characterized on α-dystroglycan, where O-mannose glycans are extended through a ribitol-phosphate linkage to form LARGE-dependent matriglycan required for laminin binding. Defects in this pathway cause congenital muscular dystrophies (dystroglycanopathies).^24^ O-Fucosylation is catalyzed by POFUT1 and POFUT2 in the ER, modifying EGF-like repeats and thrombospondin type 1 repeats, respectively. These modifications are essential for Notch signaling and related developmental pathways and can be extended by Fringe family enzymes (POFUT1 substrates), though the resulting glycan chains remain short. O-Glucosylation, mediated by POGLUT1/2/3, also targets EGF-like repeats and contributes to protein folding and quality control.^26^ O-Glucose can be further extended by xylose residues. O-Galactosylation of collagen and collagen-like domains, catalyzed by COLGALT1/COLGALT2, occurs on hydroxylysine residues and can be further modified by glucosyltransferases.^27^ O-Xylosylation initiates glycosaminoglycan (GAG) biosynthesis on proteoglycans through xylosyltransferases (XT1/2), producing the tetrasaccharide linkage region that anchors heparan sulfate and chondroitin sulfate chains.^6^

Because each class of O-glycosylation is initiated by distinct enzymes in different subcellular compartments and can be elaborated into structurally divergent glycans, no single analytical workflow can capture the full O-glycoproteome. The enrichment, fragmentation, and computational strategies discussed below reflect this fundamental diversity.

### Enrichment Methods for Different Types of Protein O-Glycosylation

Due to the low abundance of many O-glycosylated proteins, the heterogeneity of O-glycan structures, and the substoichiometric nature of individual glycoforms, enrichment is essential for global analysis of protein O-glycosylation in complex biological samples by MS-based proteomics. There are different types of protein O-glycosylation, such as O-GlcNAcylation, mucin-type O-glycosylation, and O-mannosylation, and their O-glycan structures are dramatically different. In order to target one type of protein O-glycosylation, effective enrichment methods for each type of O-glycosylation are required. In this part, we discuss the enrichment methods based on each type of O-glycosylation.

#### Protein O-GlcNAcylation

Protein O-GlcNAcylation is a highly dynamic and reversible modification, in which a single GlcNAc moiety is attached primarily to serine and threonine residues, and occasionally to tyrosine residues.^28–30^ This modification predominantly occurs in the nucleus, the cytoplasm, and the mitochondrion. It is regulated by two enzymes: O-GlcNAc transferase (OGT) and O-GlcNAcase (OGA).^30^ Since its discovery in 1984, O-GlcNAcylation has been recognized as a key nutrient and stress sensor.^30–32^ It has also been implicated in human diseases, including cancer, diabetes, and neurodegenerative disorders.^33^ Over the past decade, multiple MS-based proteomic methods were developed to investigate O-GlcNAcylated proteins, including chemoenzymatic labeling, metabolic labeling, and affinity-based approaches such as lectin chromatography, immunoprecipitation, and OGA mutant capture.^34,35^

The chemoenzymatic labeling method employs recombinant glycotransferases to selectively transfer sugar moieties onto specific glycan structures on glycoproteins. Using this approach, Torres et al. first provided evidence for protein O-GlcNAcylation by applying galactosyltransferase from bovine milk together with uridine diphosphate (UDP)-[^3^H] galactose to label the GlcNAc residue.^31^ Khidekel et al. developed an elegant method leveraging the substrate promiscuity of a mutated galactosyltransferase (B4GalT1-Y289L),^36,37^ and this engineered enzyme can selectively transfer a ketone or azide functionality onto O-GlcNAcylated proteins, enabling subsequent derivatization with biotin or fluorescent tags for detection (Figure 2A,B). This chemoenzymatic labeling method was widely used to study protein O-GlcNAcylation across different sample types, including human cells and clinical specimens.^30^ However, its implementation requires the mutated galactosyltransferase, and the functionalized sugar donors are not always commercially available. Xu et al. developed a sequential enzymatic method combining easily accessible galactosyltransferase from bovine milk and galactose oxidase (GAO) to introduce an aldehyde handle onto O-GlcNAcylated proteins, which can then be targeted by hydrazide chemistry for glycoprotein enrichment.^38^ Using this method, 251 O-GlcNAcylated proteins were identified from biological triplicates in MCF7 cells. Furthermore, employing a chemoenzymatic workflow, Hou et al. systematically analyzed protein O-GlcNAcylation on tyrosine residues in PANC-1 cells, identifying 121 O-GlcNAcylation sites on tyrosine from 93 glycoproteins.^28^ These findings underscore the power of chemoenzymatic labeling for characterizing protein O-GlcNAcylation and highlight the importance of developing novel methods to gain insights into glycobiology.

**Figure 2:**
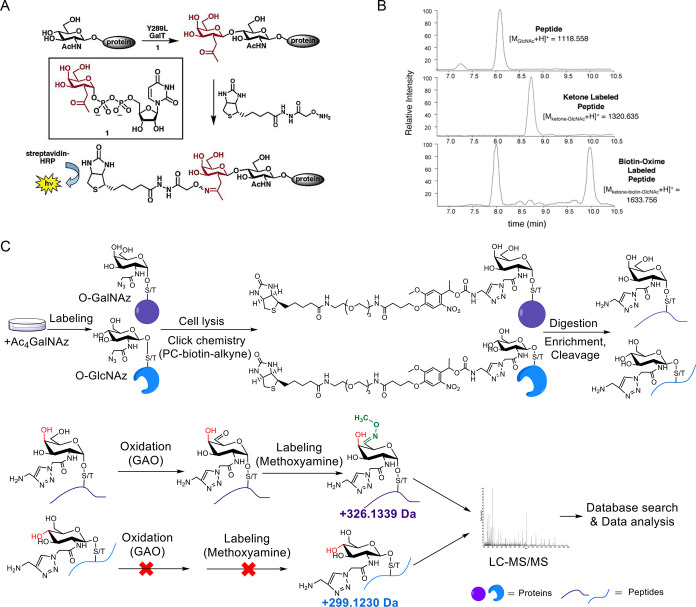
Enrichment methods for protein O-GlcNAcylation. (A) Chemoenzymatic method for detecting O-GlcNAcylated proteins. Adapted from ref 36, with permission from American Chemical Society, Copyright 2003. (B) Labeling of the peptide TAPTS­(O-GlcNAc)­TIAPG using B4GalT1-Y289L and a sugar analog. Adapted from ref 36, with permission from American Chemical Society, Copyright 2003. (C) Simultaneous enrichment and analysis of glycoproteins with O-GlcNAc and O-GalNAc. Cells were metabolically labeled with Ac_4_GalNAz, and azide-bearing glycoproteins were conjugated to PC biotin alkyne through click chemistry for enrichment. Following elution, GAO treatment allows differentiation of O-GlcNAc from O-GalNAc. Adapted from ref 57, with permission from American Chemical Society, Copyright 2022.

The metabolic labeling approach using sugar analogs was widely employed to study protein glycosylation.^39–49^ Peracetylated N-azidoacetylglucosamine (Ac_4_GalNAz) was extensively used to investigate protein O-GlcNAcylation.^50,51^ The acetal groups facilitate its passage through the cell membrane and are subsequently removed by intracellular esterases. Once inside the cell, the sugar analog is metabolically converted to UDP-GlcNAz, which can then be utilized by OGT to label proteins. The azido group serves as a bioorthogonal chemical handle that can be derivatized through reactions such as Staudinger ligation or Copper­(I)-catalyzed Azide–Alkyne Cycloaddition (CuAAC) to incorporate affinity tags, allowing for the enrichment of O-GlcNAcylated proteins.^52^

In cells, UDP-GlcNAz can be converted to UDP-GalNAz by UDP-galactose-4-epimerase (GALE), resulting in the incorporation of an azide group in both O-GlcNAcylation and O-GalNAcylation (the Tn-antigen). While the two modified groups (O-GlcNAc and O-GalNAc) have identical compositions and molecular weights, several methods have been established to distinguish them using mass spectrometry. Halim et al. systematically demonstrated that the oxonium ion patterns from the two glycans are different and can be used to distinguish O-GlcNAc from O-GalNAc, i.e., using the ratio (*m*/*z* 138 + *m*/*z* 168)/(*m*/*z* 126 + *m*/*z* 144) as a diagnostic tool.^53^ The criterion has been further applied to classify mucin-type O-glycopeptides.^54^ Furthermore, Li et al. developed HexNAcQuest, a freely accessible web tool that implements a logistic regression model using the oxonium ion ratio to reliably distinguish O-GlcNAc from O-GalNAc.^55^ This ratio has also been incorporated into database searching software such as O-Pair module in MetaMorpheus. However, this approach has some limitations. First, the oxonium ion ratio may vary with different normalized collision energies. Second, when samples are complex, particularly when glycopeptides carry extended glycan structures or when multiple glycosylation sites are present, it might be less reliable to use the ratio to infer the structures of glycans. As a result, applying this method to complex biological samples requires careful validation. Additionally, Debets et al. developed another GalNAc probe, GalNAzMe, bearing a branched N-acylamide side chain that is resistant to GALE-mediated epimerization, to selectively label O-GalNAcylated proteins without requiring GALE knockout in cells. It requires ectopic expression of an engineered pyrophosphorylase (mut-AGX1) to generate the corresponding UDP-sugar donor intracellularly.^56^

Xu et al. developed an integrative method coupling metabolic labeling, click chemistry, and an enzymatic reaction to simultaneously enrich glycopeptides with O-GlcNAc and O-GalNAc and to distinguish them through benefiting from the specificity of the enzymatic reaction (Figure 2C).^57^ Cells are first labeled with Ac_4_GalNAz, followed by click chemistry with a photocleavable (PC) biotin alkyne probe. Both O-GalNAc– and O-GlcNAc–modified peptides are enriched, and then GAO selectively oxidizes O-GalNAc but not O-GlcNAc. This generates a distinct mass shift on O-GalNAc that enables reliable differentiation of glycopeptides carrying O-GalNAc from those modified with O-GlcNAc during MS analysis. Using this method, they identified 398, 291, and 211 O-GlcNAcylated proteins and 78, 28, and 16 glycoproteins modified with the Tn antigen in Jurkat, MCF7, and K562 cells, respectively. However, metabolic labeling with peracetylated sugar analogs can induce unintended modifications on cysteine residues.^58^ To mitigate this artifact, modified analogs such as 1,6-Pr_2_GalNAz and 1,3-Pr_2_GalNAz were developed.^59^ However, unlike chemoenzymatic labeling, metabolic labeling is normally restricted to living systems.

Affinity-based approaches have long been used for the enrichment of O-GlcNAcylated proteins, with lectins playing a foundational role in the early stage of O-glycoproteomics. A key advantage of affinity-based strategies is their compatibility with native or near-native conditions across diverse sample types, including tissues, without requiring genetic manipulation or metabolic labeling. Lectins such as Wheat Germ Agglutinin (WGA) recognize terminal GlcNAc residues and enable large-scale analysis of protein O-GlcNAcylation.

Vosseller et al. first used WGA-based lectin weak affinity chromatography (LWAC) for enrichment of O-GlcNAc-modified peptides from mouse brain postsynaptic density preparations, identifying 145 unique O-GlcNAc-modified peptides and establishing this approach as a viable strategy for proteome-wide analysis of O-GlcNAcylation in neural tissues.^60^ Trinidad et al. later applied WGA-based enrichment to murine synaptosomes in a broader study characterizing both N- and O-linked glycosylation, identifying 463 O-glycopeptides on 122 O-glycosylated proteins among 453 total glycoproteins.^61^ Nagel et al. further applied lectin enrichment to identify O-GlcNAc-modified osteoblast proteins critical for bone formation.^62^ While lectin-based methods have limitations, including modest binding affinity for the monosaccharide (O-GlcNAc) and potential cross-reactivity with other terminal GlcNAc-containing glycans such as complex N-glycans, they were instrumental in advancing the field before the development of more specific tools such as anti-O-GlcNAc antibodies and engineered OGA mutants.^63^

Antibodies against O-GlcNAc, including CTD110.6, RL2, HGAC39 and HGAC85, were also developed.^64^ Yet, their utility is limited due to modest binding affinities and potential cross-reactivity with other sugar moieties.^65^ To address these limitations, Burt et al. recently generated a novel mixture of anti-O-GlcNAc monoclonal antibodies and demonstrated their effectiveness for immunoprecipitating native O-GlcNAcylated peptides from cells and tissues.^66^ These antibodies showed high sensitivity and specificity for O-GlcNAc-modified peptides, with no detectable binding to O-GalNAc or O-GlcNAc within extended glycans. Using this approach, more than 1,300 unique O-GlcNAcylated peptides and over 1,000 sites were identified from only a small amount of materials. This method was also applied to human samples, leading to the discovery of potential biomarkers of placental O-GlcNAcylation.^67^

Another innovative method employs an inactive OGA mutant (*Cp*OGA^D298N^), which retained nanomolar binding affinity to O-GlcNAcylated proteins while lacking catalytic activity.^68^ This mutant was successfully used to enrich O-GlcNAcylated substrates in vitro, enabling the identification of 52 high-confidence O-GlcNAcylated peptides on 43 glycoproteins in developing *Drosophila* embryos.^69^ More recently, Hou et al. systematically compared several affinity-based enrichment methods, including AANL6, anti-O-GlcNAc antibody and *Cp*OGA^D298N^, using the lysates of PANC-1 cells. *Cp*OGA^D298N^-based enrichment yielded 140 O-GlcNAcylated proteins, compared with 81 from the antibody-based method, and 86 from AANL6.^70^ Together, these comparisons indicate that each affinity reagent captures a distinct and incomplete subset of the O-GlcNAcylated proteome, and combinatorial or sequential enrichment strategies may be necessary to improve the overall coverage.

#### Mucin-Type O-Glycosylation

Mucin-type O-glycosylation is initiated by the addition of an α-N-acetylgalactosamine (α-GalNAc) to the hydroxyl group of serine or threonine residues, catalyzed by polypeptide GalNAc-transferases. This modification can be further extended into one of eight major O-glycan core structures through the coordinated action of multiple glycosyltransferases in mammalian cells.^23^ Mucin-type O-glycans are highly enriched on mucin domains, where the dense glycans extend the protein backbone and generate a bottlebrush-like architecture. Such extensive glycosylation protects glycoproteins from proteolysis and expands structural and functional diversity of mucins. Beyond mucins, mucin-type O-glycans are abundant on many secreted and cell-surface proteins, where they play vital roles in mediating molecular recognition, adhesion, and communication between cells and extracellular environments.^23,71^ Dysregulation of mucin-type O-glycosylation has been implicated in a wide range of human diseases, including cancer, inflammation, and congenital disorders of glycosylation.^72,73^ However, studying mucin-type O-glycosylation remains a daunting task. Unlike N-glycans with a common core structure being released enzymatically by PNGase F, O-glycans cannot be uniformly cleaved from proteins by an enzyme. In addition, the large family of GalNAc-transferases that initiate this type of O-glycosylation adds another layer of complexity. These factors have limited our understanding of the biological functions of mucin-type O-glycoproteins. To overcome these challenges, researchers have developed a variety of specialized methods for characterizing mucin-type O-glycosylated proteins.

##### Chemoenzymatic Methods

Chemoenzymatic labeling refers to in vitro modification of proteins or peptides using exogenous enzymes and activated sugar donors to selectively derivatize existing glycan structures. The Tn antigen (α-GalNAc-O-Ser/Thr), the precursor monosaccharide of mucin-type O-glycans, is a well-known tumor-associated carbohydrate antigen. Zheng et al. developed a chemoenzymatic method to selectively enrich glycoproteins bearing the Tn antigen, but not O-GlcNAc, for their global analysis by LC-MS/MS.^74^ In this method, GAO selectively oxidized O-GalNAc at the C6 position to generate an aldehyde group. The aldehyde group served as a reactive handle for capturing glycopeptides with the Tn antigen by hydrazide beads, allowing for specific enrichment of Tn-bearing glycopeptides. Enriched glycopeptides were subsequently released with methoxylamine and then analyzed by MS (Figure 3). Using the synthetic glycopeptide standards, this method demonstrated high selectivity for O-GalNAc over O-GlcNAc. Then this method was applied to analyze glycoproteins with the Tn antigen in Jurkat and MCF7 cells, respectively.

**Figure 3:**
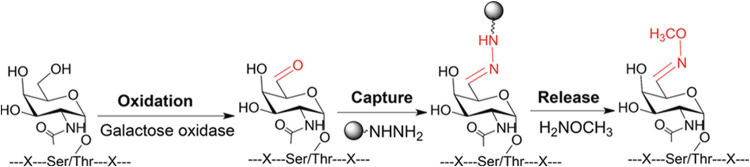
Chemoenzymatic method for specific enrichment of glycopeptides with the Tn antigen. Adapted from ref 74, with permission from John Wiley & Sons, Copyright 2017.

Another one-step enzymatic labeling method for Tn antigen detection was developed by Li et al.^75^ They synthesized novel uridine diphosphate galactose (UDP-Gal) derivatives functionalized with either a fluorescent tag or a cleavable biotin tag. These modified nucleotide-sugars serve as donor substrates for T-synthase, which transfers the tagged galactose directly onto the Tn antigen. This approach achieves direct enzymatic labeling of the Tn antigen, allowing for subsequent detection or enrichment. It was applied to identify 242 Tn-glycoproteins and 302 Tn-glycosylation sites in HEK293F^Tn+^ cells, as well as 240 Tn-glycoproteins and 330 Tn-glycosylation sites in Jurkat cells. Importantly, it is broadly applicable across sample types, highlighting its potential utility in both fundamental research and clinical samples.

Several other chemoenzymatic methods leverage human sialyltransferases for labeling the TF and related antigens. For example, Wen et al. developed a one-step labeling strategy using ST6GalNAc-IV for the sialylated TF antigen.^76^ This enzyme selectively transferred triazole-linked biotinylated sialic acid onto sialyl-TF, enabling subsequent visualization, enrichment, and global analysis of sialyl-TF glycoproteins on the cell surface. Using this method, 78 and 43 proteins were identified in MCF7 and HT29 cells, respectively.

Similarly, Aguilar et al. employed ST3Gal1 to label both the TF antigen and the core 2 O-glycans (GlcNAcβ1–6­(Galβ1–3)­GalNAcα-O-Ser/Thr) using a CMP-sialic acid-biotin donor substrate.^77^ This approach was successfully applied to screen murine tissues and human cancer tissues (lymphoma and prostate cancer). Beyond individual antigens, truncated mucin-type O-glycopeptides, including the Tn, TF, sialyl-Tn, and sialyl-TF antigens, can also be enriched using ST6GalNAc1-based labeling. For instance, Tian et al. designed a versatile “one-step probe” to simultaneously profile multiple truncated O-glycans.^78^ The probe integrated three functional modules: (1) a thermoresponsive poly­(*N*-isopropylacrylamide) (PNIPAM) polymer for enrichment without beads; (2) an ultraviolet (UV)-cleavable linker for controlled release; and (3) a sialyltransferase substrate for enzymatic labeling. Following enzymatic incorporation, glycopeptides can be captured through PNIPAM precipitation (temperature shift), released by UV cleavage, and then analyzed by MS. In total, 323 and 327 O-glycoproteins were identified from MCF7 and MDA-MB-231 cells, respectively. This approach enables global analysis of truncated mucin-type O-glycans from complex biological samples. Overall, chemoenzymatic methods offer high specificity and controlled labeling by exploiting well-defined enzyme–substrate relationships, making them particularly powerful for targeting specific glycan epitopes. Because labeling is performed in vitro, these approaches avoid perturbation of cellular metabolism and are readily applicable to primary tissues and clinical samples. However, chemoenzymatic strategies are inherently limited by the substrate specificities of the enzymes employed and are typically restricted to predefined glycan motifs, such as truncated mucin-type O-glycans. As a result, they may not comprehensively capture the full diversity of O-glycosylation present in complex biological systems and often require multiple complementary enzymes to increase the coverage.

##### Metabolic Labeling-Based Methods

In addition to exploiting glycosyltransferases directly, metabolic labeling-based methods introduce chemical reporters into glycans by feeding cells with monosaccharide precursors containing bioorthogonal chemical groups. These membrane-permeable precursors are enzymatically converted to nucleotide-sugars in cells, which are then incorporated into glycans by endogenous glycosyltransferases. The incorporated chemical handles (commonly azides or alkynes) allow for subsequent visualization, enrichment, and identification of labeled glycoproteins. The analogues of GalNAc, GlcNAc, and N-acetylmannosamine (ManNAc) are commonly used, where the native acetamide group is replaced with the bioorthogonal N-azidoacetyl or N-alkynylacetyl group.^44,79^ Feeding cells with these analogs enables metabolic incorporation of a chemical handle into glycoproteins, providing a highly versatile method for probing mucin-type O-glycosylation.

The Bertozzi group first developed the metabolic labeling method to study protein glycosylation.^80^ In 2003, Hang et al. exploited the GalNAc salvage pathway to modify mucin-type O-glycoproteins using the azido-GalNAc analogs.^46^ They synthesized several peracetylated azido-GalNAc derivatives and used flow cytometry to evaluate their incorporation efficiencies. Among them, Ac_4_GalNAz was found to be the most effective, producing a dose-dependent increase in cell-surface azide labeling with saturation at ∼50 μM. Metabolic incorporation of Ac_4_GalNAz into mucin-type O-glycoproteins introduced a bioorthogonal azide reporter, which can be tagged with phosphine- or alkyne-based probes for their detection and enrichment. This strategy specifically labeled the conserved core GalNAc residue of mucin-type O-glycans, independent of terminal glycan elaborations, allowing for proteome-wide analysis across different cell types. Importantly, the method has been extended to living animals: systemic injection of Ac_4_GalNAz to mice for labeling mucin-type O-glycoproteins in tissues (liver, kidney, and heart), serum, and splenocytes. This could even distinguish leukocyte subpopulations, demonstrating unique capabilities compared to steady-state profiling methods such as lectin blotting, lectin microarrays, or histology.^45^ The isotope-targeted glycoproteomics (IsoTaG) method was developed through the combination of metabolic labeling of O-glycopeptides with a sugar analog and tagging with a cleavable probe for glycoproteomics analysis.^81^ Collectively, metabolic labeling has become very powerful for site-specific and system-level analysis of mucin-type O-glycoproteins.

Liu et al. further used 1,6-Pr_2_GalNAz to study multiple classes of glycoproteins, including N-glycosylated, mucin-type O-glycosylated, and O-GlcNAcylated proteins.^82^ Unlike the commonly used Ac_4_GalNAz, this di-O-propionylated analog improved cellular uptake of azido sugars while minimizing nonspecific S-glyco-modification on cysteine residues.^58^ Following metabolic incorporation, the cell lysates were reacted with an alkyne–biotin probe bearing a PC linker via click chemistry, allowing for enrichment and controlled release of labeled glycopeptides for MS analysis (Figure 4). With the support of pGlyco3 software, a total of 2053 intact N-glycosites, 262 intact O-GalNAc glycosites, and 1947 O-GlcNAcylation sites were identified from the mouse lung, heart, and spleen.^83^ This method highlights the power of metabolic labeling for glycoproteomic profiling across multiple glycosylation types simultaneously.

**Figure 4:**
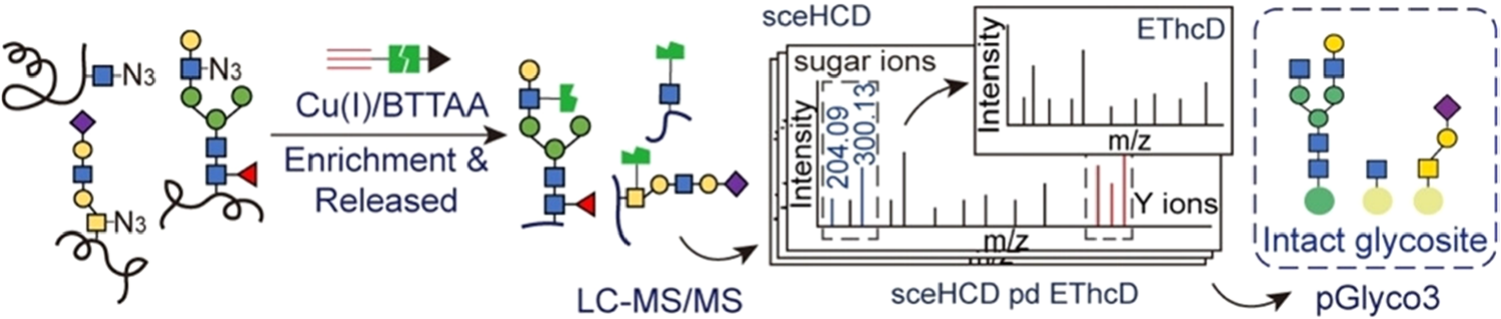
Click-iG method for simultaneous enrichment and profiling of intact N-linked, mucin-type, and O-GlcNAcylated glycopeptides: (1) Glycoproteins with glycans metabolically incorporated with azidosugars react with PC biotin alkyne through click chemistry, after which they are digested using trypsin and captured by streptavidin beads, followed by photocleavage to release the glycopeptides. (2) The click-labeled and enriched glycopeptides are analyzed by LC-MS/MS with sceHCD-pd-EThcD fragmentation. (3) The obtained raw data are analyzed by the pGlyco3 search engine. Adapted from ref 82, with permission from John Wiley & Sons, Copyright 2023.

Despite these advantages, the results from metabolic labeling may be influenced by differences in cellular metabolism and endogenous glycosylation machinery. To address such limitations, engineering glycosyltransferases provide a powerful way to expand our understanding of glycoproteins. For example, Schumann et al. developed a “bump-and-hole” variant of GalNAc transferase (GalNAcT) that selectively incorporates unnatural UDP-GalNAc analogs (e.g., UDP-GalNAz) into mucin-type O-glycans, enabling more targeted labeling of mucin-type O-glycoproteins.^84^ Cioce et al. engineered an artificial biosynthetic pathway to generate alkyne-tagged UDP-GalNAc and UDP-GlcNAc analogs from a precursor not normally accepted by the GalNAc salvage pathway.^85^ Only engineered cells harboring this pathway produced UDP-GalN6yne and UDP-GlcN6yne, which were subsequently incorporated into glycoproteins. This innovative method allows for cell-selective and tumor-specific labeling of glycoproteins, helping dissect tumor–host interactions and the roles of protein glycosylation in cancer biology.

Overall, metabolic labeling represents a versatile strategy for studying mucin-type O-glycoproteins by introducing bioorthogonal reporters into glycans. Its key advantages include the ability to enable dynamic, cell-specific, and in vivo profiling, as well as facilitating deep and sensitive identification of glycoproteins across complex biological systems. However, the introduction of non-natural monosaccharide analogs, engineered glycosyltransferases, or truncated biosynthetic pathways may influence substrate competition, enzyme specificity, and downstream glycan elaboration, potentially reshaping the native glycoproteome. The magnitude of these effects can vary substantially across cell types, metabolic states, and experimental conditions, and should therefore be carefully considered when interpreting glycoproteomic data generated using these approaches. As a result, metabolic labeling methods are particularly powerful for discovery-driven analyses and dynamic studies, but their findings are often best complemented by orthogonal enrichment strategies or validation approaches that preserve native glycosylation states.

##### SimpleCell Method

The SimpleCell (CosmcKO) technology was developed for global and site-specific analysis of protein O-glycosylation. This strategy relies on blocking the O-glycan elongation pathway in cultured cells, thereby generating “SimpleCell” lines that express homogeneous, truncated O-glycans. These simplified O-glycoproteins or O-glycopeptides can then be enriched using lectin-based methods for MS analysis. The key principle of this approach is genetic inactivation of the *COSMC* gene, which encodes an ER chaperone required for the proper folding and function of the core 1 β1,3-galactosyltransferase (C1GalT1), the enzyme responsible for initiating O-glycan elongation. Consequently, the disruption of COSMC leads to accumulation of the Tn antigen (GalNAcα-Ser/Thr) on proteins.^86^ By inactivating the COSMC gene through zinc-finger nuclease (ZFN)-mediated targeting,^87^ cells were engineered to truncate O-glycan biosynthesis to this minimal structure, enabling lectins such as *Vicia villosa* agglutinin (VVA), which specifically recognize the glycan, to be used for O-glycopeptide enrichment.^88,89^ Steentoft et al. further advanced this method by applying genetic engineering to create 12 SimpleCell lines, generating the first large-scale data set of the human O-glycoproteome with nearly 3000 glycosites identified from over 600 O-glycoproteins (Figure 5).^89^ Building on this, the Clausen group subsequently expanded the SimpleCell concept into a broader set of genetically engineered cell models, enabling more comprehensive and site-specific interrogation of mucin-type O-glycosylation. Using SimpleCell-based strategies combined with engineered reporter constructs and advanced MS workflows enabled systematic investigation of protein O-glycosylation. With these developments, the SimpleCell-based methods are very powerful not only for initial O-glycosite discovery, but also for detailed site-specific analysis of mucin-type O-glycosylation beyond the original proof-of-concept studies.^90,91^ Unlike hydrophilic interaction liquid chromatography (HILIC), which provides broad but nonspecific enrichment, or lectins, which offer targeted yet biased capture, the SimpleCell technology uniquely enables proteome-wide, site-specific mapping of protein O-glycosylation although at the cost of losing O-glycan structure information.

**Figure 5:**
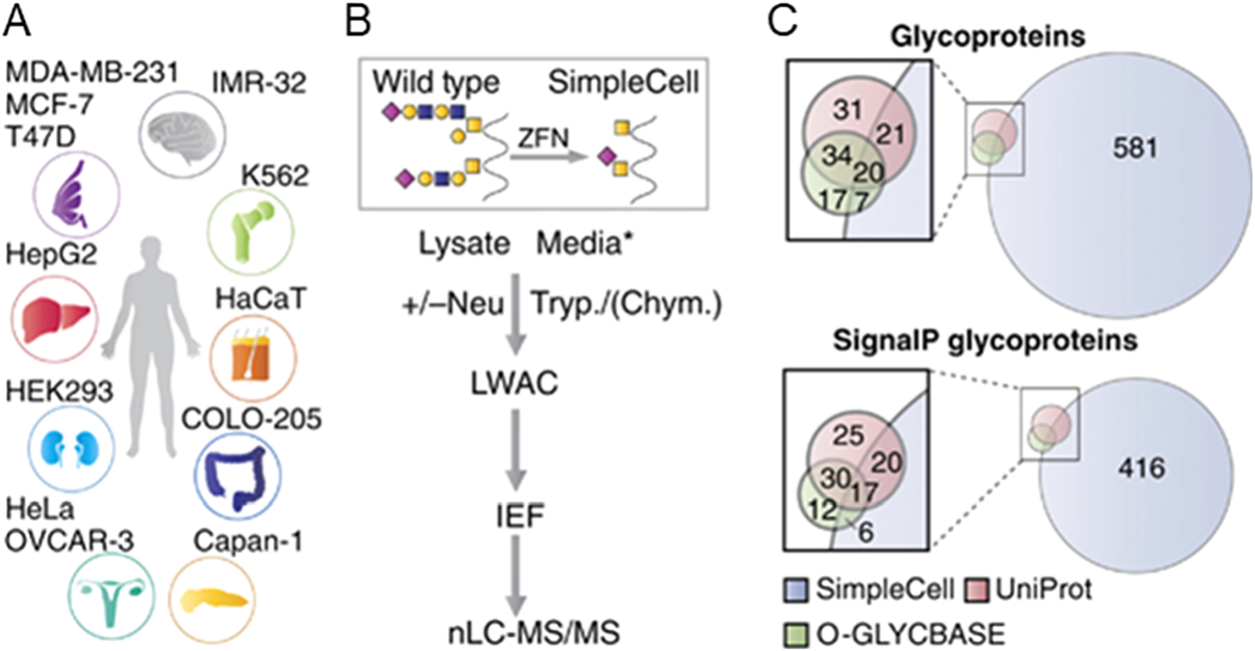
SimpleCell technology for mucin-type O-glycoproteomics. (A) SimpleCell lines originated from different organs as illustrated were generated through ZFN-mediated knockout of COSMC. (B) SimpleCells express homogeneous truncated O-glycans (Tn/STn), and glycopeptides from the cell lysates as well as conditioned media can be isolated by lectin weak affinity chromatography (LWAC) after protein digestion with trypsin or chymotrypsin. If necessary, sialic acids are removed by neuraminidase treatment. Glycopeptides are isolated on LWAC and separated by isoelectric focusing (IEF) prior to LC-MS/MS analysis. *Glycoproteins from conditioned media were preconcentrated by passing the media over a short VVA column. (C) The SimpleCell technology identified 629 glycoproteins, with only 48 in common with glycoproteins previously reported in UniProt (106 total) and O-GLYCBASE^92^ (78 total). Adapted from ref 89 Licensed under a Creative Commons Attribution (CC BY 4.0) license.

##### O-Glycoprotease-Based Methods

In addition to endogenous glycosylation enzymes, bacterial O-glycoproteases that specifically recognize O-glycans on Ser/Thr residues have emerged as powerful tools for mucin-type O-glycoprotein analysis. For example, Yang et al. employed OpeRATOR (OgpA), an O-glycoprotease derived from the mucin-degrading gut bacterium *Akkermansia muciniphila*, to develop a method termed extraction of O-linked glycopeptides (EXoO) for site-specific O-glycoproteomic analysis.^93^ The workflow consists of four steps: (i) proteolytic digestion of proteins into peptides; (ii) immobilization of peptides on a solid support; (iii) selective release of intact O-glycopeptides at glycosylation sites by OpeRATOR; and (iv) LC-MS/MS analysis. EXoO was applied to analyze O-glycoproteins in serum, and the results demonstrated that OpeRATOR cleaves N-terminal to Ser/Thr residues carrying core 1 O-glycans (TF antigen), confirming its utility for site-specific mapping of mucin-type O-glycosylation. However, OpeRATOR requires substrate desialylation for optimal activity, which limits its scope for analyzing native O-glycoproteins with sialylated glycans. To address these limitations, the methods building on the same OpeRATOR cleavage principle have been developed to improve digestion efficiency and glycosite coverage. For example, the O-glycopeptide immobilization for glycosylated peptide enrichment (O-GIG) approach combines peptide immobilization with optimized enzymatic reaction conditions, enabling more effective analysis of sialylated O-glycopeptides and extending the applicability of OpeRATOR-based workflows for site-resolved characterization of mucin-type O-glycosylation.^94^

Beyond OpeRATOR-based methods, other bacterial mucinases with distinct substrate specificities have been identified and exploited for mucin-type O-glycoprotein analysis. Malaker et al. characterized the secreted protease of C1 esterase inhibitor (StcE), a bacterial protease from *Escherichia coli* that cleaves mucin-domain glycoproteins.^21^ Using recombinant mucin-domain glycoproteins, they found that StcE recognizes a distinct consensus motif, S/T*-X-S/T, where the cleavage occurs before the second Ser/Thr only when the first Ser/Thr is glycosylated (indicated with an asterisk), guided by the recognition of both peptide and glycan. Importantly, StcE does not cleave O-GlcNAcylated or O-mannosylated peptides, but extended core 1 and core 2 O-glycans do not block its activity. StcE was also shown to digest cancer-associated mucins from cultured cells and from ovarian cancer ascites fluid, underscoring its potential for studying mucin domain structure and function. Furthermore, Shon et al. expanded the mucinase toolkit by identifying and engineering bacterial proteases with mucin-selective activity.^95^ For example, StcE^E447D^ functions as a broad mucin enrichment tool for diverse glycoforms, while BT4244^E575A^ from *Bacteroides thetaiotaomicron* is selective for truncated, asialylated core 1 O-glycans commonly associated with malignant and premalignant tissues. In addition, a catalytically inactive point mutant of StcE was found to retain binding selectivity for mucin-domain glycoproteins.^96^ Leveraging this property, a workflow using inactive point mutant of StcE (StcE^E447D^) as an affinity reagent was developed to selectively enrich mucin-domain glycoproteins from complex samples (Figure 6).^96^ StcE^E447D^ was conjugated to POROS-AL beads, and enrichment conditions, including binding time, bead-to-substrate ratio, wash buffers, and elution, were systematically optimized. Applying this workflow to cancer cell lines and ovarian cancer ascites fluid enabled the detection of hundreds of mucin-domain glycopeptides and the identification of numerous proteins not previously known to contain mucin domains. This enrichment method not only advances comprehensive analysis of mucin-type O-glycosylation but also supports translational applications, such as evaluating whether the ovarian cancer mucinome can serve as a diagnostic or prognostic indicator. More recently, the Malaker group further optimized the StcE-based enrichment workflow to improve throughput, sensitivity, and MS compatibility. Mahoney et al. introduced an MS-compatible elution strategy that replaces harsh SDS-based elution with milder detergents, enabling efficient recovery of mucin-domain glycoproteins while preserving downstream compatibility with mucinase digestion and intact glycopeptide analysis.^97^ This improved workflow substantially reduced sample input requirements and increased the mucin coverage in complex biological matrices. In parallel, Finn et al. developed GlycoFASP, a filter-based, one-pot sample preparation strategy that integrates selective O-glycoprotease digestion with molecular-weight cutoff filtration to enrich O-glycopeptides directly from complex mixtures.^98^ GlycoFASP enables flexible tuning of glycan selectivity, achieves high depletion of nonglycosylated peptides, and provides broad coverage of mucin-domain and nonmucin O-glycoproteins with minimal sample handling. Together, these advances significantly extend the utility of glycoprotease-based methods, positioning them as robust and scalable tools for comprehensive and site-resolved analysis of mucin-type O-glycosylation in both basic and translational studies.

**Figure 6:**
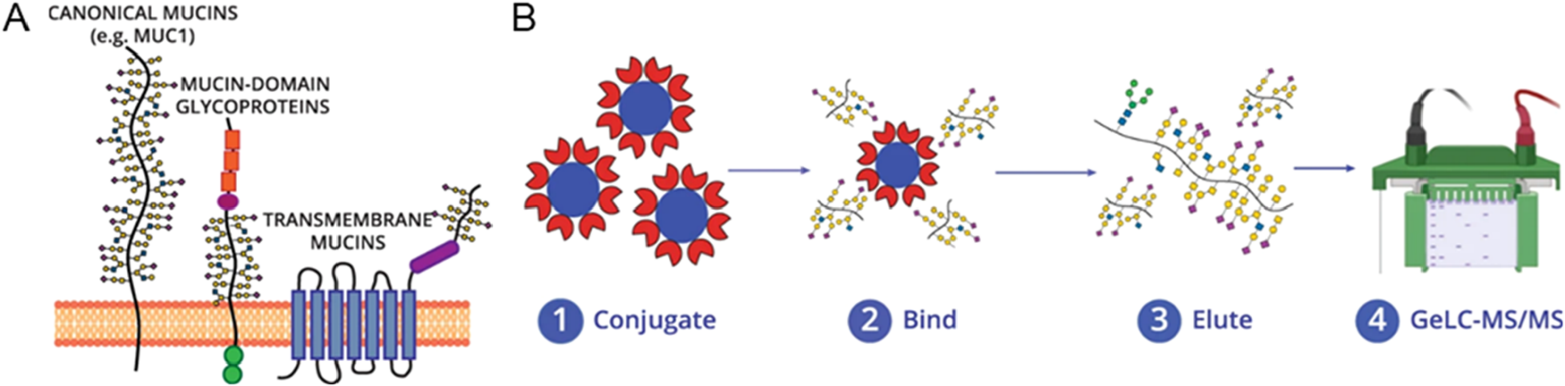
Mucinomics platform for enriching mucin-domain glycoproteins in complex samples. (A) The mucinome comprises a variety of proteins that have a densely glycosylated mucin domain. Mucin domains are found in canonical mucins, mucin-domain glycoproteins, and multipass transmembrane proteins. (B) Workflow for enrichment technique. (1) StcE^E447D^ were conjugated to POROS-AL 20 beads using reductive amination, followed by capping in Tris-HCl. (2) Complex samples (lysate and ascites) were added to the beads, (3) Elution through boiling in protein loading buffer. (4) Samples were fractionated via one-dimensional gel electrophoresis and digested in-gel using trypsin. Adapted from ref 96, Licensed under a Creative Commons Attribution (CC BY) license.

However, StcE and related mucinases primarily exhibit activity toward densely glycosylated mucin domains, limiting their broader applicability. More recently, an immunomodulating metalloprotease (IMPa) from *Pseudomonas aeruginosa* was identified as another versatile O-glycoprotease.^99,100^ Importantly, IMPa can efficiently cleave N-terminal to the Ser/Thr residues modified with a wide variety of O-glycans, including core 1, sialylated core 1, the Tn antigen, and more complex O-glycans, without the need for desialylation. Vainauskas et al. established a one-step workflow using IMPa for purified glycoproteins profiling, such as granulocyte colony-stimulating factor and receptor-type tyrosine-protein phosphatase C.^100^ In addition, Suttapitugsakul et al. applied IMPa to study the O-glycoproteome in mouse brains, and identified 695 unique O-glycopeptides from 367 O-glycoproteins, demonstrating its utility for large-scale analysis of O-glycoproteins in tissue samples.^101^

Chongsaritsinsuk et al. recently characterized another mucinase, *Serratia marcescens* Enhancin (SmE), which displayed unique activity against residues carrying highly complex glycans.^102^ SmE provides a complementary cleavage profile to StcE, targeting the N-terminus of glycosylated Ser/Thr residues. Like StcE, it can effectively cleave mucins from the cell surface, making it a valuable tool to probe mucin biology. Comparative analyses of commercial O-glycoproteases, including OgpA, IMPa, and SmE, revealed that SmE outperformed the others, yielding the most extensive coverage in terms of cleavage sites, localized glycosylation sites, glycan structures, and unique glycoforms.^102^ With the ongoing discovery and characterization of new bacterial O-glycoproteases, our capability to understand mucin-type O-glycoproteins continues to expand.

Overall, bacterial O-glycoprotease–based strategies provide a powerful and conceptually distinct approach for site-resolved analysis of mucin-type O-glycoproteins. By exploiting enzyme recognition of glycosylated Ser/Thr residues, these methods enable direct release or enrichment of intact O-glycopeptides with precise site localization, often without requiring extensive chromatographic fractionation. Moreover, the expanding repertoire of mucinases with complementary substrate specificities, including OpeRATOR, StcE, IMPa, and SmE, allows us to study diverse O-glycan structures and mucin domain architectures. However, glycoprotease-based methods also present inherent limitations. Enzyme activity and cleavage specificity are strongly influenced by glycan composition, glycan density, and local peptide context, leading to method-dependent biases for a subset of glycoproteins. As a result, glycoprotease-based strategies are most effective when applied in combination with complementary enrichment, fragmentation, and computational approaches to achieve a more comprehensive view of the O-glycoproteome.

##### Boronic Acid–Based Methods

Boronic acid–based enrichment methods rely on the formation of reversible covalent bonds between boronic acid and 1,2- or 1,3-cis-diols in the carbohydrate moieties. This interaction is highly pH-dependent: stable boronate esters form under basic conditions, while the bonds are cleaved in acidic solution (low pH), allowing for capturing glycopeptides.^103–105^ Because cis-diols are very common in glycans, boronic acid offers an unbiased enrichment strategy and avoids the inherent structural biases of traditional lectin-based methods.^106–108^ Despite that boronic acids-based methods are more established in N-glycoproteomics, it is also highly promising for characterizing the complex O-linked glycoproteome. For instance, an enrichment procedure utilizing beads derivatized with amino-phenyl boronic acid (APBA) successfully identified 36 unique MHC class I-associated peptide antigens with O-GlcNAcylation in primary leukemia samples.^109^ More recently, the deep quantitative glycoprofiling (DQGlyco) workflow using silica beads functionalized with phenyl boronic acid (PBA) allowed for the identification of 3,450 O-glycoproteins in mouse brain tissue.^110^ These applications highlight the potential of boronic acid–based methods in achieving unprecedented depth and sensitivity for O-glycoproteomic mapping.

##### Chromatography Methods

Chromatography methods are also applied for mucin-type glycoprotein enrichment, and the commonly used chromatography is HILIC.^111,112^ This approach relies on differences in hydrophilicity between glycopeptides and nonglycosylated peptides. Yang et al. exploited its effectiveness for enriching glycopeptides and found that the removal of N-glycans prior to HILIC enrichment improved the identification of O-glycopeptides by MS.^113^ They further compared two commercially available strong anion-exchange (SAX) materials, i.e., Retain AX (RAX) and Oasis MAX, to assess enrichment efficiency. Their results showed that RAX outperformed both MAX and HILIC, providing a higher yield and better specificity for O-glycopeptides. However, those chromatography methods lack the specificity for enriching mucin-type O-glycoproteins because it is based on the hydrophilicity difference between glycopeptides and nonmodified ones. But many nonglycopeptides are also highly hydrophilic. Lectin affinity enrichment provides a more targeted approach. For instance, VVA selectively recognizes the Tn antigen.^88,89^ Compared with HILIC or SAX, lectins offer improved selectivity, yet they remain constrained by their intrinsic binding specificity, relatively weak binding affinity, and potential cross-reactivity, which limit the glycoproteome coverage and biased enrichment toward certain glycoforms.

#### Protein O-Mannosylation

Protein O-mannosylation is one type of important and evolutionarily conserved glycosylation found from bacterium to human.^114^ Its biosynthesis is initiated in the ER by a family of enzymes known as protein O-mannosyltransferases. In yeast, seven such enzymes (PMT1–7) are responsible for the initiation of O-mannosylation, and their mammalian orthologs are POMT1 and POMT2. To date, POMT1 and POMT2 have been found to initiate O-mannosylation on only a limited set of human proteins, most notably α-dystroglycan.^115^

The SimpleCell technology has also been adapted to study O-mannosylation in both human cells and yeast (Figure 7A).^116,117^ In MDA-MB-231 cells, Clausen et al. used ZFN targeting of the POMGNT1 gene to simplify O-glycan structures. Following the PNGase F treatment to remove N-glycans, O-mannosylated peptides were enriched using Concanavalin A (Con A). This strategy enabled the identification of more than 50 unique O-mannosylated glycoproteins with over 230 glycosylation sites and revealed the surprising finding that the large cadherin family of membrane receptors represents a major class of O-mannose glycan carriers.^117^ The application of the SimpleCell technology in yeast uncovered that O-mannosylated proteins are annotated as nuclear, cytosolic, or mitochondrial proteins, and these cellular compartments are not typically associated with the known O-mannosylation machinery in the secretory pathway.^116^ While the SimpleCell technology effectively helps identify O-mannosylated proteins and their glycosylation sites, it does not provide information about the associated glycan structures.

**Figure 7:**
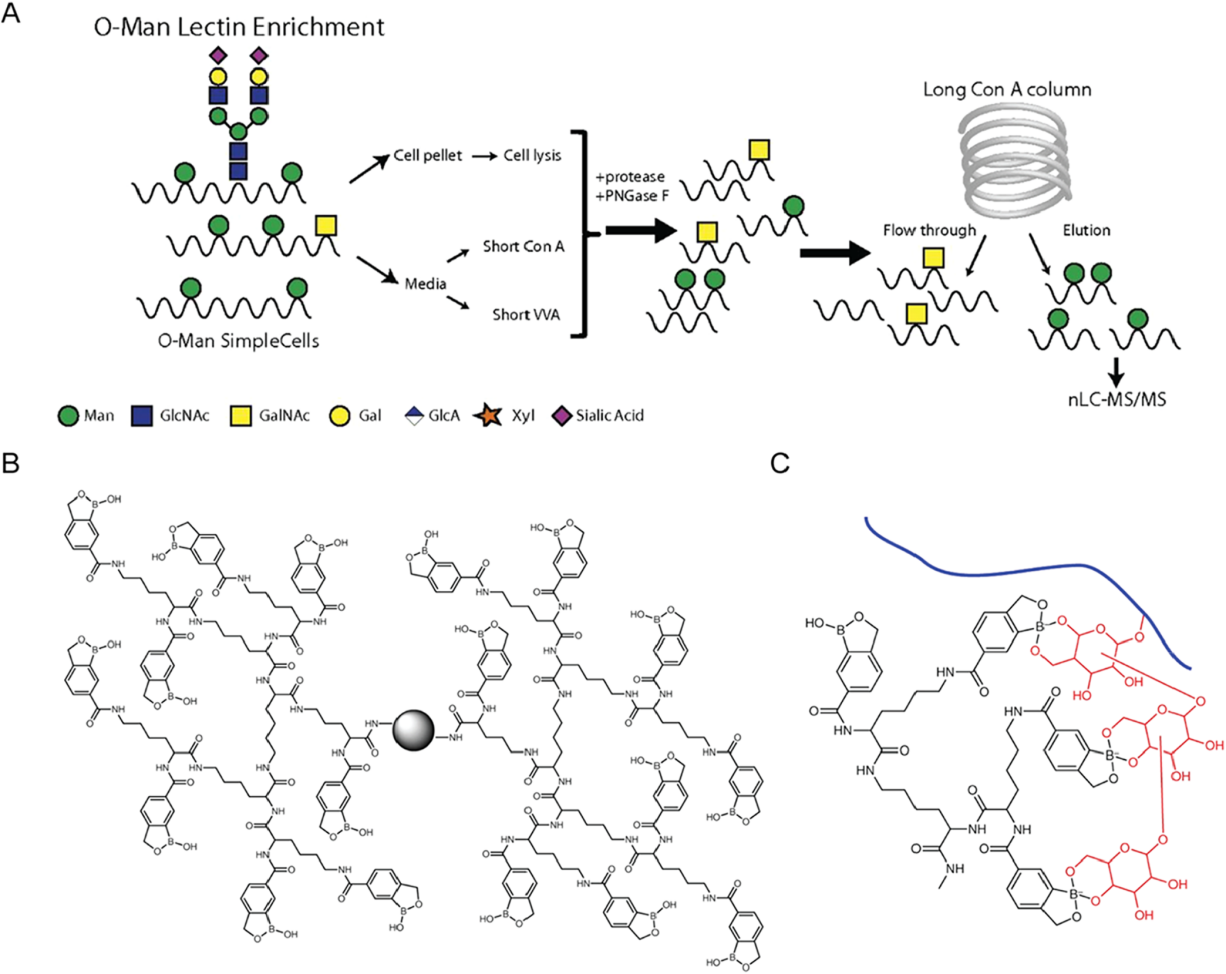
Enrichment methods for protein O-mannosylation. (A) Application of the SimpleCell technology for enriching O-mannosylated proteins. Double knockout of POMGNT1 and COSMC in MDA-MB-231 cells allows for enrichment of O-mannosylated proteins from the cell culture supernatant using either a short Con A mannose-binding lectin column or for O-GalNAc glycoprotein enrichment on a short VVA α-GalNAc-binding lectin column. Adapted from ref 117, with permission from National Academy of Sciences, Copyright 2013. (B) Structure of a dendrimer conjugated with benzoboroxole. Adapted from ref 107, with permission from Springer Nature, Copyright 2018. (C) An example of the synergistic interactions between multiple benzoboroxole groups within a dendrimer and the monosaccharide units of a single glycan on a glycopeptide. Adapted from ref 107, with permission from Springer Nature, Copyright 2018.

An alternative strategy that could potentially overcome the limitations is boronic acid–based enrichment.^106^ Xiao et al. developed a method using dendrimer-conjugated benzoboroxole to enhance the glycopeptide enrichment, leveraging synergistic benzoboroxole-glycan interactions (Figure 7B–C).^107^ In this method, glycopeptides are bound under basic conditions and subsequently released using acidic buffer. Using this strategy, 234 O-mannosylated proteins were identified in yeast. Notably, besides O-mannosylation sites, this method also allows for obtaining valuable glycan structures.

#### Protein O-Glucosylation and O-Fucosylation

Protein O-glucosylation and O-fucosylation often occur at specific domains.^6^ O-Glucosylation was originally identified on epidermal growth factor (EGF) repeats of coagulation factors and Notch (canonical O-Glc), while O-fucosylation occurs on both EGF repeats and thrombospondin type 1 repeats (TSRs).^118,119^ However, noncanonical O-glucosylation occurring on intracellular proteins has also been reported.^39,120^ In human cells, three protein O-glucosyltransferases (POGLUT1, 2, and 3) are responsible for the biosynthesis of canonical O-Glc. Among them, POGLUT1 recognizes the properly folded EGF repeats and O-glucosylates the serine residue within the consensus sequence C^1^-X-S-X-(P/A)-C^2^.^121^ Protein O-fucosyltransferases 1 and 2 (POFUT1 and POFUT2) in the ER modify EGF repeats and TSRs. Like POGLUT1, both POFUT1 and POFUT2 are exquisitely selective for properly folded substrates. O-Glucosylation and O-fucosylation of EGF repeats are essential for Notch activity. The modifications stabilize EGF repeats and facilitate proper folding of Notch in the ER, which is critical for its trafficking to the cell surface.^122^

Protein O-glucosylation on EGF repeats was unexpectedly identified using a chemoenzymatic labeling method developed for detecting protein O-GlcNAcylation (Figure 8A). This cross-reactivity likely stems from the substrate promiscuity of the galactosyltransferase from bovine milk, which can utilize both terminal glucose and GlcNAc as the substrates. Using this approach, Xu et al. identified 18 unique O-glucosylated peptides from 9 glycoproteins.^38^ In addition, several protein O-glucosyltransferases were characterized, including the *Legionella* effectors SetA and LtpM (Figure 8B).^123,124^ These enzymes recognize specific sequons and can transfer the azido-containing sugar donor to protein substrates. The introduced azido group can then be derivatized with fluorophore for fluorescence detection or with biotin for enrichment and proteomic profiling. Such glucosyltransferases serve as valuable chemical biology tools for site-specific introduction of O-Glc onto proteins of interest. However, while powerful for glycoengineering, this *in vitro* labeling does not provide insights into endogenous O-glucosylation. Recently, Zhang et al. employed a reversible chemoenzymatic labeling strategy to study both canonical and noncanonical O-glucosylation.^125^ Benefiting from the promiscuity of a mutant endoglycosidase (EndoCC-N180H), both O-Glc and O-GlcNAc can be labeled with an azido N-glycan oxazoline probe. Then, PNIPAM polymer functionalized with DBCO was conjugated to the labeled glycopeptides for their enrichment. Then enriched glycopeptides were released from the PNIPAM polymer by the treatment of wild-type endoglycosidase (EndoCC), allowing the site-specific analysis of glycoproteins with both O-Glc and O-GlcNAc by MS. Using this method, 245 O-Glc sites (147 glycoproteins) and 205 O-Glc sites (122 glycoproteins), as well as 1101 O-GlcNAc sites (361 glycoproteins) and 1288 O-GlcNAc sites (422 glycoproteins), were identified in HEK293T and HeLa cells, respectively.

**Figure 8:**
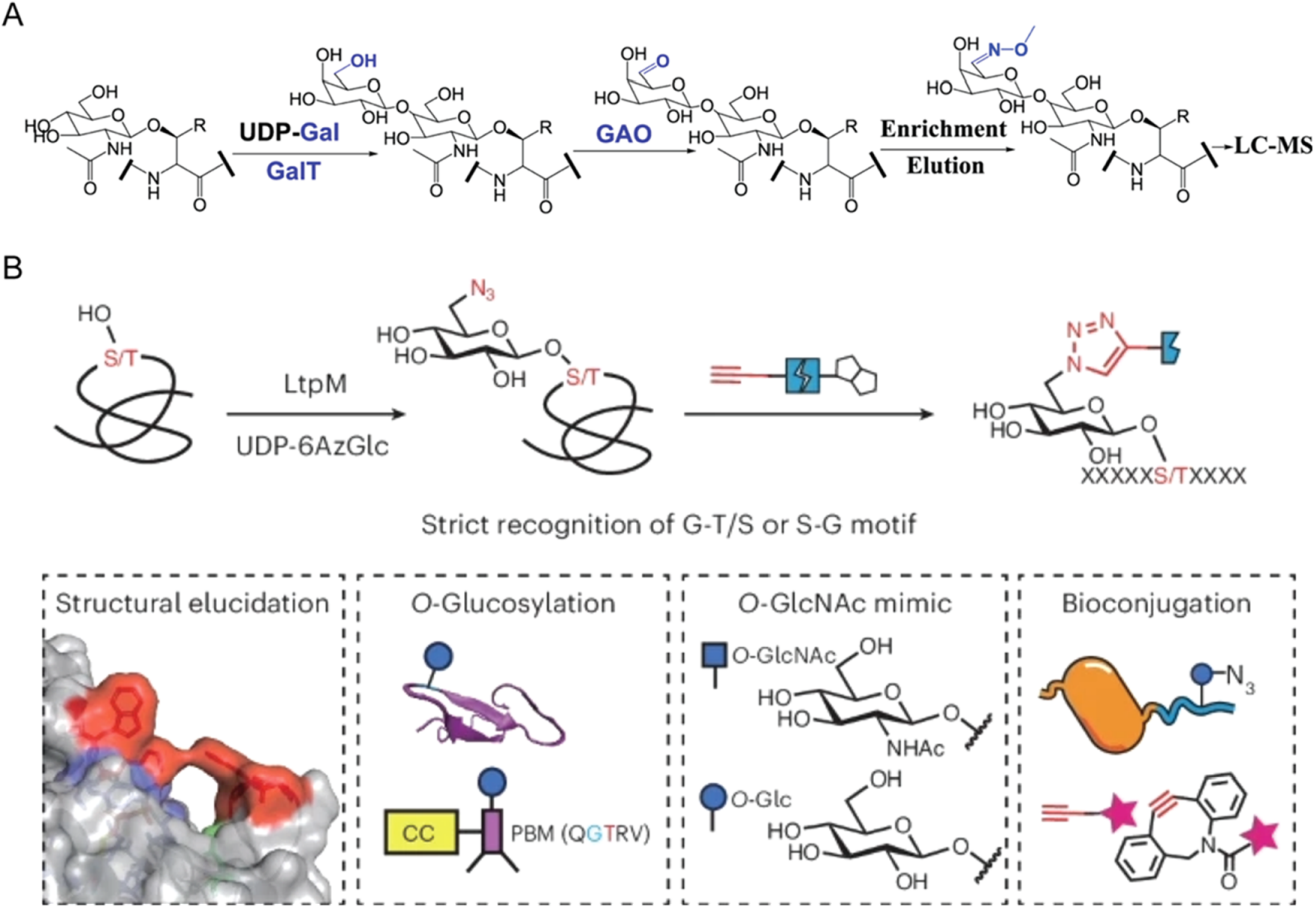
Enrichment methods for protein O-glucosylation. (A) Chemoenzymatic workflow that unexpectedly identified protein O-glucosylation. Adapted from ref 38, with permission from American Chemical Society, Copyright 2020. (B) LtpM used for site-specific O-glucosylation of diverse eukaryotic proteins. Adapted from ref 123, with permission from Springer Nature, Copyright 2025.

Metabolic labeling approaches have also been reported to identify noncanonical O-glucosylation. For example, Shen et al. observed that OGT can utilize UDP-glucose to install O-Glc onto proteins, although with 25-fold lower efficiency than using UDP-GlcNAc.^120^ Based on this finding, they fed COS-7 cells with per-O-acetyl 2-azido-2-deoxy-d-glucose (AcGlcAz), then tagged the azido-labeled proteins with alkyne–diazo–biotin. The biotinylated proteins were enriched using the streptavidin resin and subsequently released by sodium dithionite treatment. The eluted samples were subjected to SDS-PAGE and proteolytic digestion, and then analyzed by LC-MS/MS. Using this approach, 64 proteins were identified and all of them were nuclear or cytoplasmic. Similarly, Darabedian et al. found that 6-azido-6-deoxy-glucose (6AzGlc) can be converted to UDP-6AzGlc in living cells and that recombinant OGT will accept UDP-6AzGlc as a substrate *in vitro*.^39^ Furthermore, they identified an endogenous O-Glc on peptide (612–637) from host cell factor (HCF) by analyzing the proteomic data obtained using GalNAz-based IsoTaG enrichment.^126^ While powerful, the number of identified O-Glc proteins using metabolic labeling is relatively small.

Metabolic labeling is also applied to detect O-Fuc proteins. For example, Sawa et al. utilized fucose analogs combined with 1,8-naphthalimide bearing an azide or alkyne functional group to generate distinct fluorescent signals which could be observed by flow cytometry.^127^ Simultaneously, Rabuka et al. compared the effectiveness of 2-, 4-, and 6-azido fucose analogs and found that only 6-azido fucose could be used to label cell-surface glycoproteins.^128^ Furthermore, de Amo et al. microinjected zebrafish embryos with alkyne-bearing GDP-fucose to visualize the fucosylated proteins in zebrafish development for the first time.^129^ Besides, enrichment of protein O-fucosylation has been attempted using affinity-based methods involving lectins and antibodies.^130,131^ However, as noted previously, both approaches are limited by low binding affinity and insufficient specificity.

Interestingly, both protein O-glucosylation and O-fucosylation were reported to be involved in crosstalk with protein O-GlcNAcylation.^123,132^ This is expected because all types of glycosylation share the same modified residues and have similar localization either in the nucleus and the cytoplasm (O-Glc and O-GlcNAc) or on the extracellular domain (O-Glc, O-Fuc, and O-GlcNAc).^125,133^ Developing novel enrichment methods is essential to gain deeper insights into the interplay among these different types of O-glycosylation.

### Fragmentation Methods for Intact Glycopeptide Profiling

Different from protein N-glycosylation, there is no enzyme like PNGase F that can universally cleave O-glycans and generate a common tag for MS analysis. Besides, after the removal of glycans, valuable information about glycans is lost. For O-glycoproteomics studies, intact glycopeptide analysis is required to simultaneously characterize the peptide backbone, glycosylation site, and attached glycan. Collision-induced dissociation (CID) remains the most widely used fragmentation method. Under ion-trap–based CID conditions, which involve relatively slow resonant excitation and low internal energy deposition, labile glycosidic bonds preferentially dissociate, leading to extensive neutral losses of glycans and water. This dissipation of internal energy often results in limited peptide backbone fragmentation, making confident glycosylation site localization particularly challenging for O-glycopeptides. In contrast, higher-energy collision dissociation (HCD), a beam-type CID method, can generate fragments from the cleavage of both the peptide backbone (b- and y-type ions) and the glycosidic bonds (B-, Y-type ions and oxonium ions).^134,135^ Notably, fragments from HCD are typically rich in low-mass oxonium ions derived from mono- and disaccharides, which serve as diagnostic markers for glycopeptides and are widely used to trigger subsequent fragmentation events or guide glycopeptide identification workflows.^136,137^ Nevertheless, CID fragmentation generally favors cleavage of the weakest covalent bonds, resulting in spectra that are glycan-informative but often insufficient for unambiguous site localization when multiple Ser/Thr residues are present. These inherent limitations of CID-based fragmentation underscore the need for alternative dissociation methods that preserve labile glycan modifications, while providing robust peptide backbone fragmentation, motivating the development and widespread adoption of electron-based dissociation techniques such as electron transfer dissociation (ETD) for intact O-glycopeptide analysis.

#### Electron Transfer Dissociation (ETD)

ETD has become a very powerful method for intact O-glycopeptide analysis.^138^ It is a radical-driven fragmentation method in which a multiply charged peptide or glycopeptide cation captures an electron from a singly charged radical anion, leading to cleavage of the N–Cα bond and generation of c- and z-type product ions. These c/z^•^ ions that typically retain intact O-glycans are crucial for unambiguous site localization when multiple Ser/Thr residues are present in a peptide. Compared with CID, ETD provides more reliable information for site localization. Zhu et al. systematically examined O-glycopeptide fragmentation by ETD and established the rules that aid in spectral interpretation.^139^ They showed that the fragmentation patterns vary with the precursor charge state and the glycan composition. Doubly charged precursors often yield limited fragments, whereas higher charge states (≥3+) provide markedly improved sequence coverage. Importantly, ETD spectra frequently contain doubly charged c^2+^ and z^2+^ ions in the higher *m*/*z* region. Incorporating these fragments extends the sequence coverage when singly charged ions fall outside the instrument detection window. Such diagnostic features have enabled the development of algorithms that can more accurately identify O-glycopeptides and localize glycosylation sites. However, the ETD efficiency remains limited for precursors with a low charge.

#### EThcD and HCD-pd-EThcD

As discussed above, ETD alone suffers from low efficiency for O-glycopeptide analysis when the charge of precursor ions is low. To overcome this limitation, hybrid strategies such as electron-transfer/higher-energy collision dissociation (EThcD) were used for glycoproteomic studies (Figure 9).^9,140,141^ In EThcD, vibrational activation is applied immediately following ETD to promote cleavage of charge-reduced precursors, thereby enhancing fragment ion yield while retaining intact glycan moieties. This dual activation strategy generates rich c/z^•^ ions with improved sequence coverage, together with complementary glycan oxonium and Y-type ions from HCD, producing spectra highly suitable for confident site-specific assignment. Riley et al. demonstrated that EThcD consistently outperforms conventional HCD and stepped collision energy (sceHCD) for O-glycopeptides, yielding the highest rates of confident glycosylation site localization despite a slower acquisition speed.^142^

**Figure 9:**
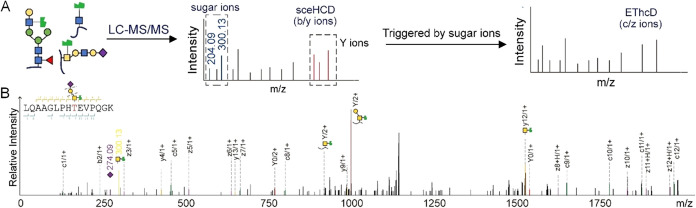
Identification of mucin-type glycopeptide using the sceHCD-pd-EThcD fragmentation method. (A) The click-labeled and enriched glycopeptides are analyzed by LC-MS/MS with sceHCD-pd-EThcD fragmentation. The presence of sugar oxonium ions in the sceHCD spectra are used to trigger EThcD fragmentation of the same precursor ions. (B) Representative MS2 spectrum from a mucin-type glycopeptide of GOLM1, which is glycosylated at T218. Adapted from ref 82, with permission from John Wiley & Sons, Copyright 2023.

To increase the fragmentation efficiency in large-scale studies, diagnostic ion-dependent methods have been reported. In HCD followed by product-dependent EThcD (HCD-pd-EThcD), an initial HCD “scout” scan detects glycan-derived oxonium ions (e.g., *m*/*z* of 204.086 or 366.139), which trigger a subsequent EThcD scan. This ensures that the time-consuming ETD-based activation is only applied to glycopeptides, but not to nonmodified ones. Liu et al. integrated sceHCD followed by EThcD (sceHCD-pd-EThcD) into the Click-iG workflow, enabling simultaneous profiling of N-, O-GalNAc-, and O-GlcNAc-glycopeptides in cells and mouse tissues (Figure 9).^82^ Similarly, Suttapitugsakul et al. used HCD-pd-EThcD for large-scale and site-specific analysis of the murine brain O-glycoproteome, identifying 221 glycosylation sites from 223 O-glycoproteins and revealing extensive sialylated core 1 glycans and the Tn antigen.^101^ Collectively, EThcD and HCD-pd-EThcD provide high-quality spectra for site-specific analysis of O-glycopeptides, and these hybrid fragmentation methods address the inherent shortcomings of ETD, enabling site mapping of densely glycosylated regions and robust O-glycoproteomic analysis.

#### Ultraviolet Photodissociation (UVPD)

UVPD developed by the Brodbelt group has emerged as a very powerful fragmentation method.^143^ Unlike electron-based methods, UVPD uses high-energy photons to fragment glycopeptides, generating extensive peptide sequence ions (a, b, c, x, y, and z) and glycan-specific fragments (A, B, C, X, Y, and Z). A key advantage is the routine production of cross-ring cleavage ions (A/X), providing linkage-specific information that is largely inaccessible to HCD or ETD-based approaches (Figure 10).^144–146^ Thus, UVPD enables both confident glycosylation site localization and in-depth structural characterization of glycans, including discrimination of isomeric linkages. Helms et al. demonstrated that coupling O-glycoprotease digestion (IMPa or SmE) with UVPD permitted comprehensive characterization of multiglycosylated peptides. This method allowed for localizing multiple adjacent O-glycosylation sites, discovering new modification sites, and resolving mucin-type glycan heterogeneity in proteins such as etanercept, TIM-1, MUC1, and MUC16.^147,148^ Compared to HCD and EThcD, UVPD provides the richest information for both peptide backbone and glycan structure, particularly when highly clustered mucin domains are analyzed. However, while UVPD generates comprehensive fragmented ions, data analysis remains a bottleneck, and advanced software tools will be very helpful to better interpret the spectra.^147,148^

**Figure 10:**
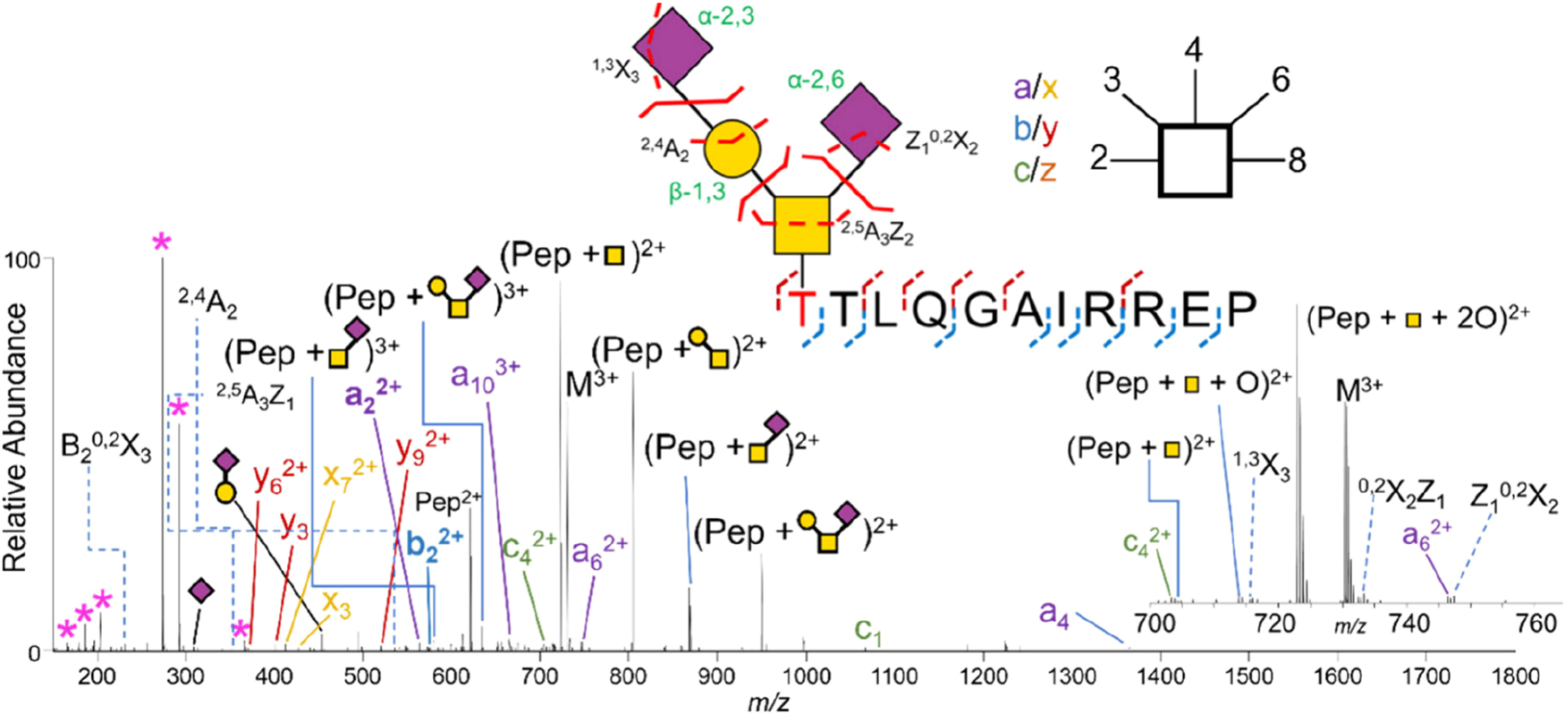
UVPD spectrum of T­[+947.23]­TLQGAIRREP (3+, *m*/*z* 730.34) from TIM-1. Representative glycosidic cleavages (solid red lines) and cross-ring cleavages (dashed red lines) are shown on the expanded glycan structure with cross-ring cleavages that confirm the linkage positions. Pink asterisks represent oxonium ions. Adapted from ref 147, with permission from American Chemical Society, Copyright 2024.

#### Electron-Activated Dissociation (EAD) and Other Fragmentation Methods

A more recent electron-driven method is electron-activated dissociation (EAD), implemented on the ZenoTOF platform.^149^ EAD is tunable, allowing for fine control of electron kinetic energy to optimize fragmentation for different analytes. Compared to CID, EAD generates more c/z ions while preserving intact glycan-peptide linkages, thereby improving glycosylation site localization. Li et al. applied EAD to analyze recombinant human erythropoietin (rhEPO), achieving simultaneous mapping of N- and O-glycosylation in a single injection.^150^ EAD preserved labile modifications such as sialylation and O-acetylation while providing improved sequence coverage and confident site assignment across multiple glycosylation sites. This approach further enabled batch-to-batch consistency assessment of rhEPO glycoforms, demonstrating the utility of EAD in biopharmaceutical quality control. Moreover, Macauslane et al. showed that although CID yielded more glycopeptide identifications overall, EAD increased site localization confidence.^151^ Combining EAD with CID (EAciD) enhanced both the number and quality of identifications, offering a balanced solution for comprehensive glycoproteomics.^151^ Despite its advantages, EAciD presents several limitations. Specifically, its parameters need to be tuned to get optimized identification and site localization results for different types of O-glycosylation samples. Additionally, the low intensity of the EAD-fragmented ions may decrease the confidence of the identification results.

Recent work highlights the benefit of combining complementary fragmentation methods. For example, Luo et al. developed GlycoTCFM, which employs two independent LC-MS/MS acquisitions, i.e., EThcD-sceHCD and sceHCD, to analyze HILIC-enriched, de-N-glycosylated peptides.^152^ Unlike HCD-pd-EThcD, in which EThcD is conditionally triggered only when oxonium ions are detected in an initial HCD scout scan, in GlycoTCFM, EThcD-sceHCD and sceHCD were used as two independent MS/MS acquisitions, so that all precursors are fragmented by both methods. In the EThcD-sceHCD acquisition, EThcD and sceHCD alternate within a duty cycle, generating c/z-type backbone ions that preserve the intact glycan moieties for confident site localization. The standalone sceHCD acquisition provides complementary glycan-informative fragments (oxonium ions and Y-ion series) and b/y peptide backbone ions, yielding higher scan speed and additional glycopeptide identifications. The integration of the two data sets substantially increases the coverage: in human semen samples, 69 intact O-glycopeptides were uniquely identified by EThcD-sceHCD and 68 by sceHCD alone, with 190 shared between the methods. This integrative strategy revealed distinctive O-glycoproteoform signatures in cancer-associated proteins, illustrating the power of multifragmentation workflows to resolve biological heterogeneity in the O-glycoproteome.

In summary, no single method provides a complete solution for very complex glycopeptide samples. ETD and EThcD remain gold standards for reliable O-glycosylation site localization, UVPD offers unrivaled structural insights into glycans, and EAD provides an alternative with translational potential. The future of O-glycoproteomics lies in integrated workflows combining these dissociation methods to achieve comprehensive characterization of the O-glycoproteome at both the peptide and glycan levels.

### Quantitative Characterization of Protein O-Glycosylation

Quantitative glycoproteomics is critical to help understand glycoprotein functions in biological systems.^153,154^ By integrating quantitative strategies with different enrichment approaches and fragmentation methods for protein O-glycosylation, numerous studies have established workflows to measure the changes of O-glycosylated proteins across different conditions and biomedical samples.^155,156^

Stable isotope labeling with amino acids in cell culture (SILAC) is a straightforward and powerful approach for quantifying proteins and glycoproteins.^157^ Qin et al. combined metabolic labeling with SILAC to establish a quantitative, time-resolved workflow for investigating the dynamics of O-GlcNAcylated proteins.^49^ Using this method, they quantified the turnover rates for 533 O-GlcNAcylated proteins in NIH 3T3 cells and found that ∼14% were very stable. Despite its simplicity, SILAC has the following limitations: expanding its multiplexing capacity is challenging, and the resulting spectral complexity complicates data analysis, restricting its utility for studies involving multiple conditions. A key limitation of SILAC is its restriction to cultured cells, rendering it incompatible with clinical specimens such as patient tissues or biofluids.

Introducing isotopically labeled groups onto peptides or proteins through chemical reactions has also been applied for quantitative O-glycoproteomics. For instance, by integrating the SimpleCell technology with dimethyl labeling using light and medium formaldehyde, Narimatsu et al. assessed the contributions of individual GALNTs to the O-glycoproteome in human HEK293 cells.^158^ In another example, Liu et al. employed an isotopic photocleavable tagging approach to quantitatively investigate protein O-GlcNAcylation.^159^ They designed and synthesized an isotopically labeled photocleavable linker, which incorporated into a chemoenzymatic labeling workflow to quantify protein O-GlcNAcylation in colorectal cancer metastasis. Coupled with HCD-pd-EThcD fragmentation method, this strategy enabled quantification of 625 high-confidence O-GlcNAc sites in SW620 and SW480 cells.

The tandem mass tag (TMT) method has been extensively applied in multiplexed proteomics. By integrating metabolic labeling with TMT, Xu et al. developed a workflow to systematically investigate the distribution and dynamics of protein O-GlcNAcylation in the nucleus and the cytoplasm of human cells (Figure 11A).^51^ The result showed that O-GlcNAcylated proteins in the cytoplasm are generally more stable than those in the nucleus. Furthermore, when combined with protein solubility analysis, this workflow was applied to examine the role of O-GlcNAcylation in regulating protein solubility and its impact on RNA-protein condensates.^32^ Unexpectedly, the findings revealed that O-GlcNAcylation globally decreases protein solubility. A specific group of O-GlcNAcylation events were found to promote the dissociation of RNA-protein condensates under heat stress, while some enhanced the formation of RNA-protein condensates during the recovery phase. Using site mutagenesis, inhibition of O-GlcNAc transferase, and fluorescence microscopy, they further validated that O-GlcNAcylation regulates the formation of biocondensates for YTHDF3 and NUFIP2. Yang et al. developed a workflow integrating EXoO with TMTpro 16-plex to investigate GalNAc-polypeptide: N-acetylgalactosaminyltransferase (GALNT) 2-specific O-glycosylation sites across diverse mouse tissues.^160^ Glycopeptides were enriched from protein extracts using the EXoO protocol and subsequently analyzed by LC-MS/MS employing the HCD-pd-EThcD method. The resulting raw data were searched using MSFragger-Glyco, pGlyco3 and O-Pair, leading to the identification of 2,154 O-glycosylation sites from 595 glycoproteins. This workflow was further applied to characterize the GALNT3-specific O-glycoproteome in mouse (Figure 11B).^155^

**Figure 11:**
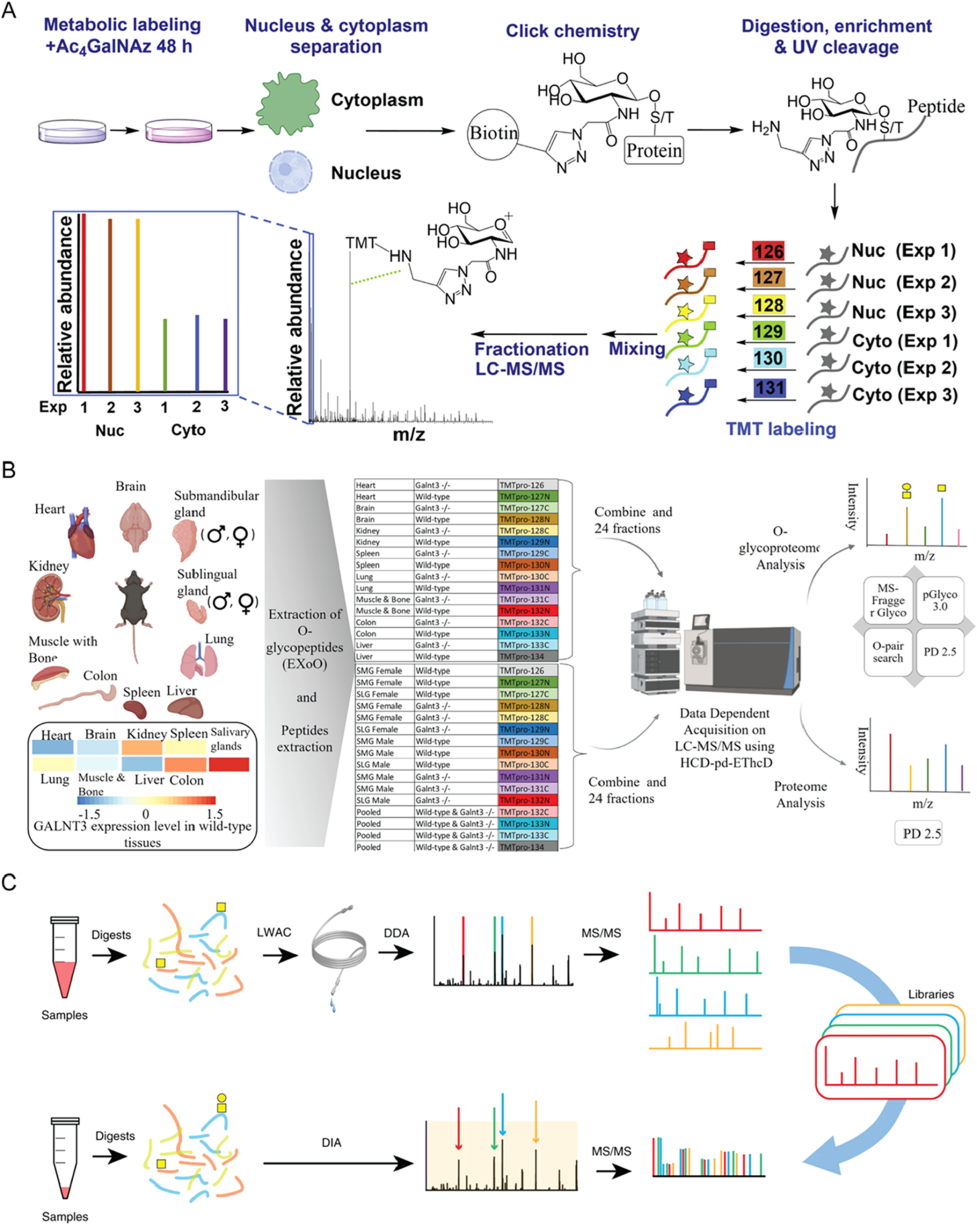
Quantitative analysis of protein O-glycosylation. (A) Quantification of O-GlcNAcylated proteins distributions in the nucleus and the cytoplasm. Adapted from ref 51, with the permission from Elsevier, Copyright 2022. (B) In vivo mapping of the mouse GALNT3-specific O-glycoproteome. Adapted from ref 155, with the permission from Elsevier, Copyright 2024. (C) Workflow for generating DIA glycopeptide libraries and applying Glyco-DIA for glycoproteomic analysis. Adapted from ref 161, with the permission from Springer Nature, Copyright 2019.

Label-free quantification (LFQ) has also been applied in O-glycoproteomic research.^162^ By combining EXoO with LFQ, Yang et al. quantified aberrant O-glycoproteins associated with human kidney tumor, identifying 56 O-glycoproteins with significant differences between the normal and tumor samples.^93^ More recently, Zhao et al. introduced a data-processing strategy that integrates glycoform-specific database searching, reference library-based MS1 feature matching, and MS2 identification propagation, facilitating rapid, in-depth, and reproducible LFQ of protein O-glycosylation across a large cohort of human urine samples.^163^ Using this workflow, 1,300 O-glycopeptides corresponding to 435 O-glycoproteins were identified from 36 urine samples.

In addition to data-dependent acquisition (DDA), data-independent acquisition (DIA) has gained increasing popularity due to its robustness in achieving deep peptide identification and quantification in proteomics.^164^ While DIA was successfully applied to N-glycoproteomic analysis with promising results, its application in O-glycoproteomics remains at an early stage.^8,165^ A study by Vakhrushev et al. introduced the Glyco-DIA approach, in which high-quality DIA spectral libraries were constructed (including the Tn-DIA and T-DIA libraries), using the SimpleCell lines and wild-type cell lines/human serum, respectively (Figure 11C).^161^ A single-shot analysis of human serum with the Glyco-DIA library, combined with LFQ, enabled the identification and quantification of 269 distinct glycopeptide sequences carrying up to 5 different core 1 O-glycans from 159 glycoproteins. However, extending Glyco-DIA to samples with more complex O-glycans will require additional high-confidence O-glycopeptide spectral library. Future work may include additional experiments and machine learning-based prediction.^166,167^

### Computational Analysis of Intact O-Glycopeptides

While experimental advances in enrichment and fragmentation have substantially expanded O-glycopeptide detection, computational analysis remains a critical bottleneck in the field. Several search engines now support intact O-glycopeptide identification, broadly divided by algorithmic strategy. Glycan-first tools such as pGlyco3 index Y-complementary ions to identify glycan compositions before peptide assignment, achieving rigorous control of the glycan-level false discovery rate (FDR) through a spectrum-based decoy approach.^83^ Peptide-first tools, including MSFragger-Glyco and the O-Pair module within MetaMorpheus, exploit the preferential cleavage of glycosidic bonds under collisional activation: HCD spectra provide peptide backbone fragment ions and Y-ion series that constrain total glycan composition, while paired EThcD spectra retain intact glycans on c/z-type backbone fragments, enabling site-specific localization. O-Pair introduced a graph-based algorithm that is approximately 2,000-fold faster than Byonic, along with a four-tier localization confidence system (Levels 1, 1b, 2, and 3) that has become a widely adopted reporting framework.^168^ Byonic treats glycans as variable modifications, making it computationally prohibitive for multiply O-glycosylated peptides and potentially susceptible to elevated false-positive rates at the glycosite level.^169^ More recent entrants include PEAKS GlycanFinder, which integrates deep-learning-based glycan de novo sequencing with database searching, though its published benchmarking has focused primarily on N-glycopeptides.^170^ Protein Prospector has also been updated with improved glycoform reporting depth and was among tools evaluated in a recent comparison of different types of software for O-glycopeptide identification.^171^ A fundamental challenge is the combinatorial search space: multiple O-glycan compositions distributed across dense Ser/Thr sites can generate an enormous number of candidate glycoforms per peptide, rendering the database searching space intractable at the proteome scale. Peptide-first and paired-scan strategies circumvent this combinatorial explosion but limit the ability to identify unanticipated glycan compositions.

FDR estimation for glycopeptides presents another challenge. Peptide-only target-decoy approaches, as implemented in early versions of some search engines, leave glycan assignments unvalidated. Entrapment experiments revealed substantial glycan-level error rates under the peptide-only FDR control. For example, significant proportions of erroneous NeuAc-containing glycan assignments were reported in yeast, an organism that does not produce sialylated glycans.^83^ The field increasingly recognizes that joint peptide-and-glycan FDR, as implemented in pGlyco3 and recent MSFragger-Glyco updates, is essential for reliable glycopeptide identification.^172^

O-Glycan site localization presents additional difficulties because no consensus sequon restricts candidate sites, and every Ser and Thr residue must be considered. Mucin domains with high density of these residues make site localization even more challenging. HCD-only data typically achieves confident site localization for approximately 8–15% of O-glycopeptide spectrum matches, compared with approximately 65% when EThcD is employed, making electron-based dissociation much more favorable for site-resolved O-glycoproteomics.^142^

A more recent comparison of different types of software by Hogan et al. highlighted this challenge: despite using experimentally matched glycan databases to eliminate library composition as a confounding variable, each software returned a substantial proportion of glycopeptides unique to itself, with limited concordance among them.^173^ The authors concluded that glycoproteomic analyses should employ multiple types of software combining peptide-first and glycan-first strategies to increase identification confidence. Deep learning models for glycopeptide fragment ion and retention time prediction have advanced rapidly but currently almost exclusively target N-glycopeptides; the first extension to O-glycopeptides has only recently been reported.^174,175^ Until robust benchmarks and standardized reporting guidelines are established, the pragmatic recommendation is to employ orthogonal search strategies, require EThcD data for site localization, and apply joint peptide-glycan FDR control.

## Conclusions and Perspectives

MS-based O-glycoproteomics has advanced substantially over recent years. With the development of novel enrichment methods and effective fragmentation techniques such as ETD, EThcD and UVPD, quantitative analysis of intact O-glycopeptides has become increasingly feasible.^176^ Applying these methods to study diverse biological samples will further expand our understanding of the properties and functions of O-glycosylated proteins.

Despite remarkable progress on MS-based O-glycoproteomics, there are still some challenges for systematic and accurate analysis of O-glycosylated proteins in complex biological samples. The structural complexity and heterogeneity of O-glycans, together with the typically low abundance of many O-glycoproteins, continue to hinder confident identification and quantification of O-glycosylated proteins at the systems level. Reliable site localization is further complicated by the lack of consensus sequence motifs and the frequent clustering of multiple glycosylation sites within short peptide regions.

It is worth noting that the majority of O-glycosylation sites reported in large-scale glycoproteomics studies are based on fragments in the tandem MS with FDR control, rather than individually experimentally validated. Nevertheless, an increasing number of studies have begun to bridge the gap between site identification and biological function. For example, Griffin et al. developed NOTISE, a systems-level approach that integrates O-GlcNAcylation site identification with OGT interactome mapping and protein–protein interaction network analysis, to reveal functional and regulatory relationships of O-GlcNAcylation, demonstrating how large-scale glycosylation sites identified from MS-based proteomics can be translated into biological insights.^177^ In the field of phosphoproteomics, Ochoa et al. addressed a similar challenge by developing a phosphosite functional score that integrates 59 features, spanning MS evidence, evolutionary conservation, regulatory context, and structural environment, to systematically prioritize functionally relevant phosphosites from large-scale data sets.^178^ As the size of O-glycosylation sites continues to grow, similar computational frameworks that incorporate features such as glycosylation site conservation, local structures, and tissue-specific occupancy may help prioritize sites for targeted functional validation and accelerate the translation of O-glycoproteomics discoveries into mechanistic understanding.

Furthermore, data analysis remains a major bottleneck, as current software tools show limited overlap in glycopeptide identifications and often lack robust false discovery rate (FDR) estimation.^173^ Integrating intact O-glycopeptide identification with quantitative strategies such as TMT labeling greatly expands the search space, often at the expense of time and computational cost. Continued development of highly efficient algorithms is required to achieve robust identifications and accurate quantification within reasonable time scales. Novel algorithms and robust FDR control will be critical to improve accuracy, reproducibility, and confidence in O-glycoproteomics studies.

AI and deep learning are poised to transform multiple stages of the O-glycoproteomics workflow. The recent development of deep learning models for glycopeptide spectral prediction, as discussed above,^174,175^ opens new opportunities for improving O-glycoproteomics analysis. Once mature, these models may improve database searching sensitivity by enabling spectral library prediction for glycoforms and could assist in distinguishing true from false identifications through rescoring frameworks. Beyond spectral prediction, machine learning approaches hold promise for automated annotation of glycopeptide fragments and glycan structures directly from mass spectra, which will serve as an independent means of validating the search results for glycopeptide identification, site localization, and glycan structure assignment. Currently, such validation requires extensive manual inspection and is highly labor-intensive. Dedicated software tools for automated quality assessment will significantly accelerate O-glycoproteomics data analysis.

In addition, while ETD is commonly used for O-glycosite localization, the overall low number and abundance of c/z ions generated often limit site localization confidence. Hybrid EThcD improves c/z ion coverage through supplemental collisional activation; however, increasing the supplemental activation energy also promotes cleavage of labile glycosidic bonds, resulting in partial glycan loss that compromises glycan structure assignment. This represents an inherent limitation of currently available fragmentation approaches for O-glycopeptide analysis, arising from the competing fragmentation requirements of the peptide backbone and the glycosidic bonds. Novel dissociation strategies or alternative analytical approaches that can simultaneously preserve intact glycans while achieving comprehensive backbone fragmentation remain to be developed for more confident site localization and glycan structure elucidation.

Rapid advances in MS instrumentation are expected to significantly expand our understanding of O-glycoproteins. New MS platforms such as Astral and Astral Zoom, with faster scanning speed and high resolution, enable deeper O-glycoproteome coverage with improved sensitivity through HCD-based workflows. However, these platforms currently lack electron-based dissociation capabilities, which are essential for confident O-glycosylation site localization. Additionally, other platforms such as ZenoTOF and Omnitrap are also very powerful for glycoproteomics, and they provide alternative fragmentation capabilities, including electron-activated dissociation and flexible multistage fragmentation schemes, which are well suited for site-resolved analysis of intact glycopeptides.^151,179^ At the same time, chemical and biological innovations, including proximity labeling approaches,^180,181^ CRISPR-based glycoengineering, and advanced imaging strategy,^182^ offer powerful ways to investigate the functional roles of O-glycoproteins in their native cellular contexts. Moreover, the ability to analyze O-glycoproteins from clinical samples, such as tissues and liquid biopsies,^183^ opens promising opportunities for translational research with direct applications in precision medicine.

Looking forward, integrating O-glycoproteomics with other omics such as genomics, transcriptomics, lipidomics, and metabolomics will enable systems-level understanding of glycobiology in health and disease. While analytical advances have greatly improved structural elucidation, deciphering how O-glycosylation modulates protein folding, trafficking, interactions, and stability will require synergy with complementary approaches, including gene editing, glycosyltransferase perturbation, and spatial proteomics. Another promising direction is to map the spatial distribution of O-glycoproteins across tissues and within cellular compartments. Together, these developments underscore the growing potential of O-glycoproteomics, not only to advance fundamental glycobiology but also to contribute to precision medicine through the discovery of novel biomarkers and therapeutic targets.
